# Insurance Churn and the COVID-19 Pandemic

**DOI:** 10.1001/jamahealthforum.2025.1467

**Published:** 2025-06-27

**Authors:** Sarah P. Shubeck, Emily Crawford, Matthew J. Notowidigdo

**Affiliations:** 1 Department of Surgery, Biological Sciences Division, University of Chicago, Chicago, Illinois; 2 Booth School of Business, University of Chicago, Chicago, Illinois

## Abstract

**Importance:**

Many individuals in the US face a high risk of losing their health insurance coverage and experiencing insurance churn, especially those enrolled in Medicaid. Prior research has found that the risk of losing insurance coverage remains high in the US even after the Patient Protection and Affordable Care Act significantly reduced the number of uninsured individuals.

**Objective:**

To estimate whether the Families First Coronavirus Response Act (FFCRA) decreased insurance churn.

**Design, Setting, and Participants:**

This study used 2 quasi-experimental approaches: an interrupted time series approach and a difference-in-difference approach. Both approaches use individual-level data from the Medical Expenditure Panel Survey from January 2015 to December 2022. In the difference-in-difference analysis comparing individuals with Medicaid to individuals with private health insurance coverage, the probability that insured individuals aged 2 to 64 years lose insurance over the next 12 months before and after the FFCRA was estimated. Data were analyzed from January to November 2024.

**Main Outcomes and Measures:**

Primary outcome was the share of insured individuals who lost insurance coverage over the next 12 months. This measure was defined for all insured individuals and defined separately for individuals with Medicaid and for individuals with private insurance coverage.

**Results:**

The sample included 96 473 individuals. Of these, 46 779 (49.7%) were male, and the mean (SD) age was 31.9 (18.1) years. In the interrupted time series analysis, the FFCRA was associated with a reduction in insurance churn by 2.06 percentage points (β = −0.021; 95% CI, −0.024 to −0.018; *P* < .001). In the difference-in-difference analysis, the FFRCA reduced Medicaid churn by 5.51 percentage points (β = −0.055; 95% CI, −0.060 to −0.050; *P* < .001). Combining these estimates, 65.0% (95% CI, 54.8-75.3) of the reduction in insurance churn came from the reduction in Medicaid churn.

**Conclusions and Relevance:**

In this study, the FFCRA was associated with a significantly decreased risk of losing health insurance. Without the FFCRA, an estimated 2.94 million individuals with Medicaid would have lost insurance coverage each year during the COVID-19 public health emergency.

## Key Points

**Question:**

Did the Families First Coronavirus Response Act (FFCRA) reduce insurance churn?

**Findings:**

In this interrupted time series analysis and difference-in-difference analysis of a nationally representative survey sample including 96 473 individuals, the FFCRA significantly reduced insurance churn, with most of the reduction in insurance churn coming from the reduction in Medicaid churn caused by the FFCRA.

**Meaning:**

The FFCRA significantly decreased the risk of losing health insurance for individuals enrolled in the Medicaid program.

## Teaser

This study estimates whether the Families First Coronavirus Response Act decreased insurance churn.

## Introduction

Many individuals in the US face a high risk of losing their health insurance coverage and experiencing insurance churn, which is the short-term loss and gain of insurance coverage due to factors such as administrative requirements or changes in eligibility.^1^ Since being enacted in March 2010, the Patient Protection and Affordable Care Act (ACA) has substantially reduced the rate of uninsurance in the US, from 20% to 12.5%.^2^ However, previous research has found very little change in the probability of losing health insurance coverage after the ACA.^2^ As a result, insurance churn remains stubbornly persistent in the US, especially for those on Medicaid.

In fact, Medicaid enrollees experience the highest rates of insurance churn. Annually, approximately 10% of Medicaid-enrolled individuals experience a gap in coverage of less than a year, most often due to the procedural requirements of completing the recertification processes to verify income, family status, and residence.^3^ While some individuals lose Medicaid coverage due to true loss of eligibility, most enrollees have their coverage reinstated within 12 months after initial coverage loss indicating unchanged eligibility.^1^ This coverage churn can result in higher health care system costs, greater administrative expenses, and gaps in care receipt.^4^ These lapses in coverage can also result in transitions in covered clinicians or health systems, which can lead to potential care disruptions or delays in essential treatments.^5^

In the early months of the COVID-19 pandemic, Congress passed the Families First Coronavirus Response Act (FFCRA) in April 2020. For the first time, the nationwide adoption of the FFCRA created a continuous enrollment condition that immediately halted Medicaid disenrollments. Because of this, many individuals were able to remain on Medicaid without having to recertify or reenroll. As a result, the FFCRA would be expected to ultimately reduce insurance churn, although the precise magnitude of the reduction is uncertain. Therefore, we sought to evaluate the association between the FFCRA and reductions in insurance churn overall and Medicaid churn in particular using 2 complementary research designs: an interrupted time series (ITS) analysis and a difference-in-difference (DID) analysis. We are aware of 2 prior studies of insurance churn during the COVID-19 pandemic using the same data that we analyze in this article, and we find broadly similar results to both studies.^6,7^ We extend the previous literature in 3 main ways. First, we introduce 2 quasi-experimental research designs to estimating the association between the FFCRA and reductions in insurance churn, allowing us to draw stronger inferences than the prior work. Second, we assess how much of the overall reduction in insurance churn comes from a reduction in Medicaid churn. Lastly, we provide novel and transparent graphical evidence using an event study analysis, which provides support for the validity of the FFCRA as a natural experiment.

## Methods

### Data Sources and Study Population

We used the publicly available survey data from the Integrated Public Microdata Series Medical Expenditure Panel Survey (MEPS).^8^ The MEPS is a large-scale survey of individuals and health care professionals across the US, sampled from respondents of the National Health Interview Survey and nationally representative of the noninstitutionalized US civilian population. The MEPS interviews roughly 15 000 households each year. Each year, a new cohort is added and is typically surveyed over 5 rounds of interviews roughly equally spaced over 2 calendar years.

The MEPS survey collects information on the scope, cost, and availability of health insurance and the frequency, type, costs, and funding sources of health services used. The survey collects data on individuals’ insurance coverage for each month in the 2-year survey window. We begin our sample in January 2015 (1 year after the start of the ACA) and extend the sample to December 2022 because the unwinding of the FFCRA’s continuous coverage provision began in 2023.^9,10^ All reported statistics and empirical analysis use the annual survey weights provided, which represent the inverse probability of selection into the sample, adjusted for nonresponse with poststratification adjustments for age, race and ethnicity, and sex using the US Census Bureau’s population totals. Race and ethnicity were self-reported and categorized according to MEPS codes.^11,12^

For each cohort, we restrict the sample to individuals who responded to all 5 survey waves and were aged 2 to 64 years at the end of the second survey year, following the sample restrictions in prior work using the MEPS to study insurance churn.^2^ The resulting sample is 96 473 unique individuals. This study was reviewed by University of Chicago Institutional Review Board and determined to be exempt. We followed the Strengthening the Reporting of Observational Studies in Epidemiology (STROBE) reporting guideline.

### Outcomes

Our key variable of interest is derived from the individual’s insurance coverage. This is reported monthly based on interview answers, which ask the respondents about their health insurance coverage each month over the reference period (typically the previous 4 to 5 months). In our main analysis, we define insurance churn as the share of individuals beginning a given month insured and then experiencing at least 1 month uninsured at any point in the following 12 months.

### Exposure

At the onset of the COVID-19 pandemic, the US Department of Health and Human Services declared a public health emergency, and Congress passed the FFCRA, which was enacted in April 2020. In exchange for increased federal support for a state’s Medicaid system, the act created a new continuous enrollment condition, which temporarily halted Medicaid disenrollment.^13^ This continuous enrollment provision was adopted by all US states. The FFCRA was an unprecedented change to the Medicaid program; importantly for our DID research design, the FFCRA did not include any policy changes that directly affected the enrollment or eligibility of individuals with private health insurance coverage.

### Statistical Analysis

We used an ITS model to compare changes in insurance churn before and after the FFCRA was enacted in April 2020. The ITS model assumes that absent the FFCRA that insurance churn would have followed the same linear time trend as in the years prior to the enactment of the FFCRA. That is, any structural break in the time trend after the FFCRA was implemented will be interpreted as being caused by the FFCRA. Because our primary outcome is losing insurance coverage over the next 12 months, we modeled 2 structural breaks in the time series: one 12 months before the FFCRA was enacted and one when the FFCRA was enacted. For inference, we clustered standard errors by year-month.

The ITS model imposes strong assumptions, and previous researchers have raised concerns about the reliability of extrapolating linear trends to analyze health policies.^14^ These concerns are especially relevant for the FFCRA, since it was enacted during the early months of the COVID-19 pandemic. As a result, we also used a DID model to estimate changes in insurance churn before and after the FFCRA was enacted, comparing individuals with Medicaid to individuals with private insurance coverage. The DID model assumes parallel trends between treatment (Medicaid) and control (private insurance) samples. If individuals with Medicaid and private insurance were otherwise affected similarly by the COVID-19 pandemic, then the DID estimates will isolate the effect of the FFCRA on insurance churn. We conducted the DID analysis using an event study model that includes year-month fixed effects, unit (treatment/control) fixed effects, and interactions between Medicaid indicator and each year-month (excluding the month 13 months before the FFCRA was implemented as a normalization for identification).^15^ For inference, we clustered standard errors by year-month.

All analyses were conducted using Stata SE version 16.1 (StataCorp). *P* values were calculated using Student *t* tests, and all *P* values were 2-tailed. Significance was set at *P* < .05. Data were analyzed from January to November 2024.

### Heterogeneity Analysis and Sensitivity Analysis

We separately estimated the ITS model for every major demographic group defined by sex (male and female), age (younger than 18 years, aged 18 to 44 years, and aged 45 to 64 years), education (high school or less and some college or more), and race and ethnicity (Hispanic, non-Hispanic Black, and non-Hispanic White; due to small sample sizes, American Indian or Alaska Native, Asian, Native Hawaiian or Other Pacific Islander, and multiple race categories were excluded). We also estimated results separately by income (above or below 200% of the federal poverty level), employment, and self-reported health status. A primary goal of our heterogeneity analysis was to assess whether the groups with higher rates of Medicaid enrollment also experienced the largest reductions in insurance churn.

In our sensitivity analysis, we assessed robustness to controlling for age, race and ethnicity, sex, and education. We also modified our primary insurance churn measure to be any loss of insurance coverage over the next 6 months (instead of 12 months), and we report results excluding those that are only partially affected by the continuous coverage provisions. We also verified that our results are robust to using all other insured individuals (not on Medicaid) as an alternative control group for the DID analysis and to defining insurance churn by requiring 2 consecutive months of uninsurance.

## Results

### Sample Characteristics

The sample included 96 473 individuals. Of these, 46 779 (49.7%) were male, and the mean (SD) age was 31.9 (18.1) years; 28 947 (20.4%) were Hispanic, 16 030 (13.0%) were non-Hispanic Black, 41 378 (56.4%) were non-Hispanic White, and 10 118 (10.2%) were another race (including American Indian or Alaska Native, Asian, Native Hawaiian or Other Pacific Islander, and multiple races). We present summary statistics for our sample in Table 1. Most respondents in the pre-FFCRA and post-FFCRA cohorts were White, privately insured, and had at least a high school education. Medicaid enrollees comprised 18% of survey respondents. Our adult pre-FFCRA sample skewed slightly younger, with a greater proportion of sampled individuals aged 18 to 44 years compared with 45 to 64 years. About 37% had a high school degree or General Educational Development equivalency credential as their highest degree completed, and the remainder had at least some college education. The post-FFCRA sample had broadly similar demographic characteristics.

**Table 1.** Summary Statistics and Balance Tests

Characteristic^a^	Mean (SE), %			*P* value for balance tests^b^
	Total	Pre-FFCRA	Post-FFCRA	
Total, No.^c^	96 473	82 852	13 621	NA
Sex				
Female	50.3 (50.0)	50.4 (50.0)	50.0 (50.0)	<.001
Male^d^	49.7 (50.0)	49.6 (50.0)	50.1 (50.0)	
Age group, y				
<18	27.1 (44.5)	27.3 (44.6)	26.4 (44.1)	<.001
18-44	42.5 (49.4)	42.4 (49.4)	43.0 (49.5)	<.001
45-64	30.4 (46.0)	30.3 (46.0)	30.6 (46.1)	<.001
Race and ethnicity group^e^				
Hispanic	22.7 (41.9)	22.4 (41.7)	24.0 (42.7)	<.001
Non-Hispanic Black	14.5 (35.2)	14.4 (35.1)	14.7 (35.5)	<.001
Non-Hispanic White	62.8 (48.3)	63.2 (48.2)	61.2 (48.7)	<.001
Highest level of education^f^				
High school or less	37.0 (48.3)	37.1 (48.3)	36.4 (48.1)	<.001
Some college or more	63.1 (48.3)	62.9 (48.3)	63.6 (48.1)	<.001
Health insurance coverage				
Medicaid	19.6 (39.7)	19.3 (39.5)	20.6 (40.5)	<.001
Private employer	55.9 (49.6)	56.1 (49.6)	54.8 (49.8)	.81
Other	10.2 (30.3)	10.0 (30.0)	10.9 (31.2)	<.001
Uninsured	17.8 (38.3)	17.9 (38.3)	17.5 (38.0)	<.001

Abbreviations: FFCRA, Families First Coronavirus Response Act; NA, not applicable

^a^ We present an alternate version of these summary stats broken out by coverage type in the eAppendix in Supplement 1. See eTable 1 (any insurance), eTable 2 (Medicaid), eTable 3 (private insurance), eTable 4 (other coverage), and eTable 5 (uninsured) in Supplement 1.

^b^ *P* values from balance tests on differences between the pre-FFCRA and post-FFCRA periods. We cluster standard errors by year-month.

^c^ Sum of unique Medical Expenditure Panel Survey identity indicators. All statistics are calculated among individuals aged 2 to 64 years.

^d^ Age groups, sex, education levels, and insurance coverage are defined according to the response when first observed in the sample.

^e^ Race and ethnicity were self-reported and categorized according to Medical Expenditure Panel Survey codes.^11,12^ Due to small sample sizes, American Indian or Alaska Native, Asian, Native Hawaiian or Other Pacific Islander, and multiple race categories were excluded. In cases of discrepancy, race and ethnicity classification is defined as the modal response.

^f^ Statistics on educational attainment are calculated only among individuals 25 years or older in the beginning of the survey.

Of those who reported being insured in the first survey month, about one-half had private health insurance provided by an employer or union, about one-fifth had Medicaid, and about 3% had insurance through the ACA private exchanges. The remaining had insurance through other forms of public coverage, such as Medicare coverage due to disability or TRICARE.

**Figure 1.**
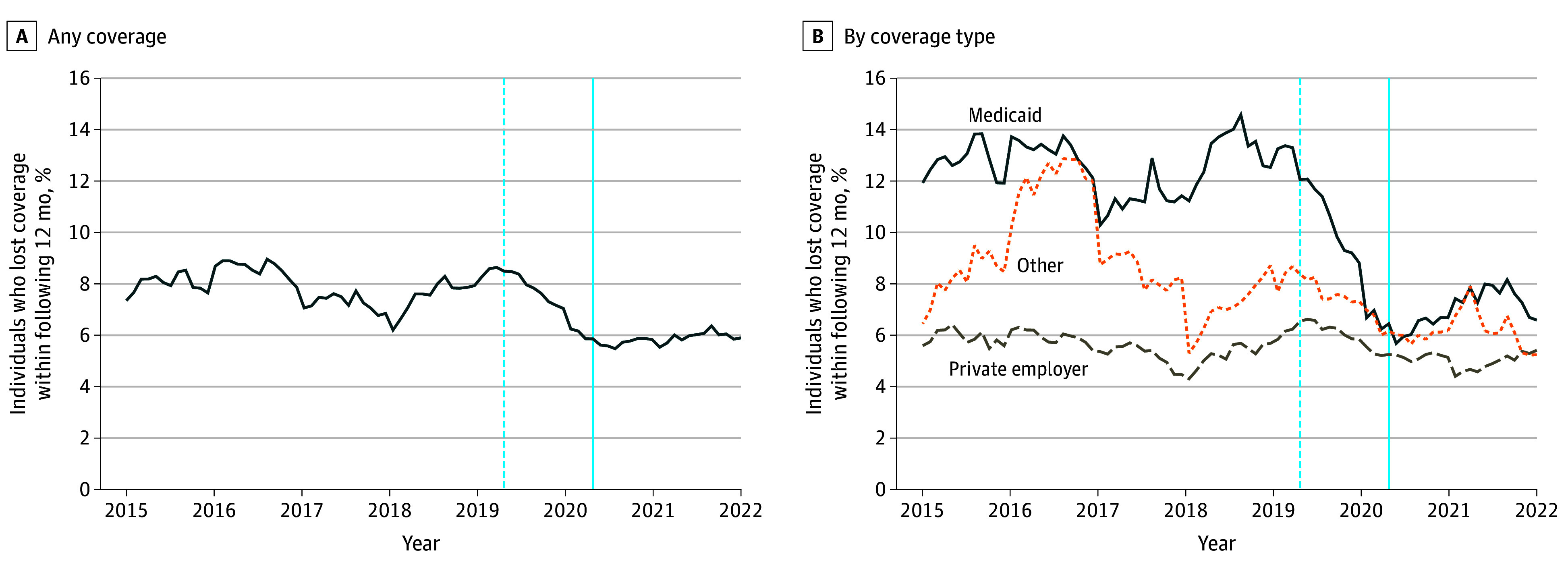
Share of Individuals Who Lost Coverage Within the Following 12 Months (Raw Data) Among those with health insurance coverage, the share who ever experienced a period of being uninsured in the following 12 months is shown. The vertical solid blue line indicates the date on which the Families First Coronavirus Response Act was enacted (April 2020). The vertical dashed blue line shows the beginning of the partially treated sample—cohorts where future coverage overlaps with the passage of the Families First Coronavirus Response Act.

Figure 1 shows that in the pre-FFCRA sample period, Medicaid enrollees experienced churn at a rate of 12.6%, more than 50% greater than the rate of those with private, employer-based coverage, at 5.6%. Prior to the enactment of the FFCRA, rates of churn across demographic groups were broadly similar (Table 2).

**Table 2.** Baseline Rates of 12-Month Insurance Churn by Demographic Group

Characteristic^a^	Individuals who lost coverage, %			
	Any coverage^b^	Medicaid	Private coverage (employer provided)^c^	Other coverage^d^
All individuals^e^	7.9	12.6	5.6	8.9
Sex				
Female	7.9	12.9	5.3	8.5
Male	7.9	12.3	5.9	9.3
Age group, y				
<18	6.6	9.1	4.2	7.5
18-44	10.4	19.4	7.3	13.1
45-64	6.0	11.5	4.5	6.2
Race and ethnicity group^f^				
Hispanic	10.6	13.3	7.9	11.2
Non-Hispanic Black	9.8	12.0	7.8	8.0
Non-Hispanic White	6.7	12.6	4.8	8.5
Highest level of education^g^				
High school or less	10.0	14.7	7.8	7.2
Some college or more	6.6	15.5	5.0	9.3

^a^ Displays the preperiod rates of 12-month churn by demographic group and insurance type. We define the preperiod as all times before future coverage overlaps with the passage of the Families First Coronavirus Response Act (ie, January 2015 up to the partial treatment period).

^b^ Any coverage is comprised of individuals with any health insurance coverage, including private coverage (from employer or union group, from other group or nongroup insurance, from a federal or state exchange, or from an unknown source) and public coverage (Medicare, Medicaid, or TRICARE).

^c^ Private coverage indicates coverage from a private employer or union group.

^d^ Other coverage is all coverage under the any coverage group, excluding Medicaid and private, employer-provided coverage.

^e^ Statistics are calculated among individuals aged 2 to 64 years. Age groups, sex, education levels, and insurance coverage are defined according to the response when first observed in the sample.

^f^ Race and ethnicity were self-reported and categorized according to Medical Expenditure Panel Survey codes.^11,12^ Due to small sample sizes, American Indian or Alaska Native, Asian, Native Hawaiian or Other Pacific Islander, and multiple race categories were excluded. In cases of discrepancy, race and ethnicity group are defined as the modal response.

^g^ Statistics on educational attainment are calculated only among individuals 25 years or older in the beginning of the survey.

### ITS Estimates

**Figure 2.**
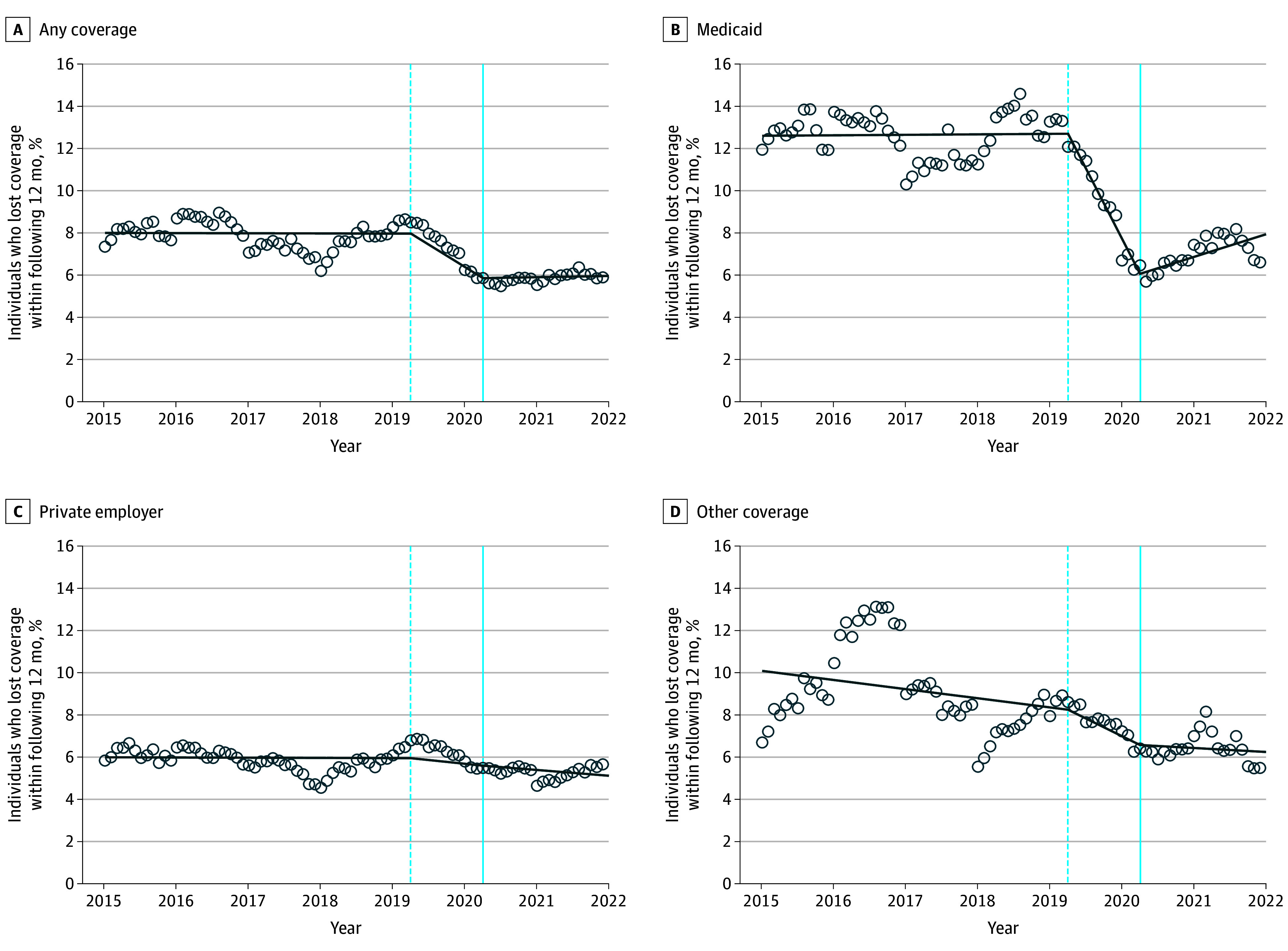
Share Who Lost Coverage Within the Following 12 Months The circles indicate data for each calendar month, among those with any insurance coverage (Medicaid, private employer-provided coverage, or other coverage), the share who ever experienced a period of being uninsured in the following 12 months. The solid lines are constructed by fitting a linear spline to the shares, allowing for separate slopes in the pretreatment, partial treatment, and treatment periods. The vertical solid blue line indicates the date on which the Families First Coronavirus Response Act was enacted (April 2020). The vertical dashed blue line shows the beginning of the partially treated sample—cohorts where future coverage overlaps with the passage of the Families First Coronavirus Response Act.

Following the passage of the FFCRA, the overall rate of insurance churn declined substantially, as shown in Figure 1. The same figure also shows a significant reduction in the rate of churn among Medicaid enrollees. Figure 2 reports the results from the ITS analysis, displaying the fitted values from the regression model on top of the insurance churn means for each year and month. The ITS model results are reported in eTable 6 in Supplement 1 and imply that the FFCRA was associated with a decrease in insurance churn by 2.06 percentage points (β = −0.021; 95% CI, −0.024 to −0.018; *P* < .001). Figure 2 shows that there was an overall decrease in insurance churn starting right around the time of the FFCRA, and the large reduction in churn was concentrated among Medicaid enrollees. There was no significant decrease in insurance churn for individuals with private insurance; churn rates for this subpopulation remained very stable during the entire sample period.

Overall, the ITS model provides clear visual evidence of a sharp reduction in insurance churn, primarily driven by a reduction in Medicaid churn. While the ITS model fits the data well, the model relies on strong assumptions. In particular, any other effects of the COVID-19 pandemic on insurance churn that are not directly caused by the FFCRA can bias estimates of the effects of the FFCRA on insurance. Because of this, we complemented the ITS analysis with a DID analysis that compared Medicaid enrollees to the privately insured.

### DID Estimates

**Figure 3.**
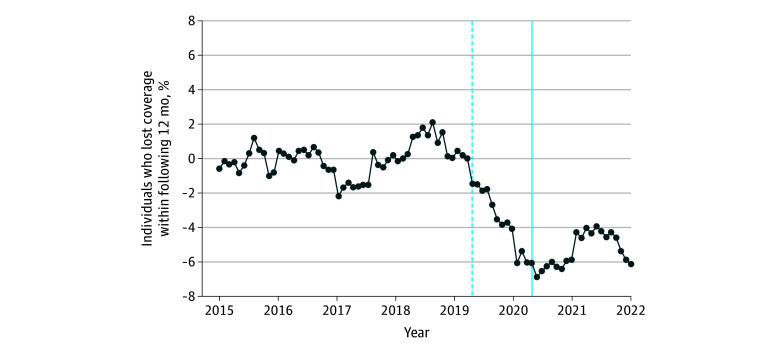
Difference-in-Difference Estimates of the Effect of the Families First Coronavirus Response Act on Insurance Churn The dots indicate the normalized difference-in-difference coefficient, where the outcome variable is 12-month churn. The treatment group is comprised of all those with Medicaid coverage, and the comparison group is all those private coverage from an employer or union group. We present an alternate version where we define the Medicaid comparison group as those with any health insurance coverage other than Medicaid, including private coverage (from employer or union group, from other group or nongroup insurance, from a federal or state exchange, or from an unknown source) and public coverage (Medicare or TRICARE) in eFigure 10 in Supplement 1. The vertical solid blue line indicates the date on which the Families First Coronavirus Response Act was enacted (April 2020). The vertical dashed blue line shows the beginning of the partially treated sample—cohorts where future coverage overlaps with the passage of the Families First Coronavirus Response Act.

We report the DID estimates in Figure 3. We found very similar trends in insurance churn between those enrolled in Medicaid and privately insured individuals in each of the years prior to the FFCRA. As soon as the FFCRA was enacted, the Medicaid churn rate decreased relative to those with private insurance, and the difference stabilized for the remainder of the sample period. When we estimate a DID specification that collapses the event time dummies into 2 post-FFCRA dummies (one for the 12 months prior to the enactment of the FFCRA and one for the entire post-FFCRA period), we estimate that the FFCRA was associated with a reduction in Medicaid churn by 5.51 percentage points relative to individuals enrolled in a private, employer-sponsored insurance coverage (β = −0.055; 95% CI, −0.060 to −0.050; *P* < .001).

We combined the DID estimates with the ITS estimates to assess the role of Medicaid churn in accounting for the overall decline in insurance churn. To do this, we took the DID estimate and multiplied it by the share of the overall insured population on Medicaid prior to the FFCRA (24.3%), which gives a predicted decline in overall churn of −1.34 percentage points (95% CI, −1.46 to −1.23). The ITS estimates indicate a 2.06 percentage point (β = −0.021; 95% CI, −0.024 to −0.018; *P* < .001) decrease in churn in the insured population, suggesting that the reduction in Medicaid churn accounts for roughly 65.1% (95% CI, 54.8-75.3) of the overall reduction in insurance churn. These results imply that the decrease in insurance churn after the FFCRA was enacted comes primarily from the reduction in Medicaid churn. We estimate that 53.3 million individuals were on Medicaid in the year prior to the FFCRA in our sample. If we multiply this number by the DID estimate, we estimate that 2.9 million individuals on Medicaid would have lost insurance coverage each year during the COVID-19 public health emergency without the FFCRA.

### Heterogeneity Analysis and Sensitivity Analysis

The continuous coverage provisions established by the FFCRA explicitly targeted Medicaid beneficiaries. Therefore, among demographic groups with higher rates of Medicaid enrollment, we expected larger declines in insurance churn. We estimated group-specific ITS models (eFigures 1 to 4 in Supplement 1) for each demographic group (defined by age, education, race and ethnicity, and sex) and find that groups with higher rates of Medicaid enrollment (eg, those younger than 18 years, Hispanic subgroups, and non-Hispanic Black subgroups) also experienced the largest reductions in insurance churn. We also found larger reductions for individuals with lower income and similar reductions by employment status and self-reported health status (eFigures 5 to 7 in Supplement 1).

In our sensitivity analysis, we modified our primary insurance churn in 2 ways. First, we modified the measure to be any loss of insurance coverage over the next 6 months (instead of 12 months) and found similar results (eTable 8 and eFigure 11 in Supplement 1). Second, we verified that our results are robust to defining insurance churn as experiencing 2 consecutive months of uninsurance (eFigure 8 in Supplement 1), which may reduce measurement error in the insurance churn measure.^16^ Additionally, we verify that we found similar results to excluding all insurance churn data covering the first 12 months of the COVID-19 pandemic to avoid any confounding effects of the early stages of the pandemic as well as disruptions to the typical MEPS data collection procedures (eFigure 9 in Supplement 1).^17^ We also found very similar results using unweighted data instead of the annual survey weights (eFigures 12 to 14 and eTables 9 and 10 in Supplement 1) and to controlling for age group, sex, race and ethnicity, and education group (eFigures 15 to 17 and eTable 11 in Supplement 1). Lastly, in our main DID analysis, we compared Medicaid enrollees to the privately insured, but we found similar results using all other insured individuals as a control group instead (eFigure 10 and eTable 7 in Supplement 1).

## Discussion

We present 2 quasi-experimental approaches to estimate the impact of the FFCRA on health insurance churn. As described above, the FFCRA and the COVID-19 public health emergency significantly reduced insurance churn for all insured individuals. Most notably, we found that the decrease in insurance churn was primarily due to a reduction in Medicaid churn likely directly attributable to the FFCRA. This demonstrates that the FFCRA succeeded in a way that the ACA did not—significantly decreasing the probability of losing health insurance coverage.

Prior to the FFCRA, Medicaid enrollees often experienced instability in health insurance coverage. Our estimates suggest that the FFCRA was associated with reductions in Medicaid churn to rates much closer to that of privately insured populations. This may have mitigated health care access challenges among Medicaid enrollees during the COVID-19 public health emergency period of 2020 to 2023.

While the FFCRA provisions and recertification changes resulted in less stringent reenrollment procedures overall, there was likely still substantial state-level variation in the effects of the FFCRA, as well as variation across states prior to the FFCRA due to differences in Medicaid policy across states (eg, Oregon already had a continuous enrollment provision for all children younger than 5 years prior to the FFCRA). We used the publicly available MEPS data, which does not have state-level indicators, but future work will use the restricted-use MEPS data with state identifiers to investigate state-level variation in Medicaid churn prior to the FFCRA as well as state-level heterogeneity in the effects of the FFCRA. The impact of the FFCRA may have varied across Medicaid expansion states and nonexpansion states and interacted with preexisting state policies as well (such as Medicaid outreach efforts).

The results of this study demonstrate that the FFCRA has created a unique opportunity to assess if reducing Medicaid churn can improve the quality of care, health care utilization, and the wellness of the Medicaid-enrolled population. Mitigating coverage loss may have improved the timeliness and guideline concordance of care delivery, which could potentially reduce long-standing health disparities. Additionally, since insurance churn often results in delayed or deferred preventive care, the FFCRA may have improved the quality of care and reduced costs as well. While there is substantial literature documenting the positive impacts of health insurance coverage on an individual’s health, mental well-being, and consumer finances, there is much less work on the effects of insurance churn on these same outcomes.^18,19,20^

### Limitations

Our study has limitations. First, both quasi-experimental approaches require assumptions that may not hold exactly. The ITS analysis can be confounded by other policies occurring around the same time as the FFCRA. The DID analysis is robust to confounding national policies as long as the policies affect Medicaid enrollees and individuals with private insurance coverage similarly. While acknowledging these limitations, we are reassured that our main results are robust to different samples, specifications, and control groups. Second, we used self-reported survey data, which creates scope for measurement error in our primary insurance coverage variable. Future research should replicate our main results using administrative data on insurance coverage and combine survey and administrative data to assess how individuals perceive continuous coverage. Third, we interpreted our results as suggestive of a causal effect of the FFCRA on insurance churn, but we are not able to determine the precise mechanisms through which the effects of the FFCRA occurred. We suspect that the FFCRA decreased insurance churn primarily through the continuous enrollment provision and the temporary relief from recertification requirements, but future research should investigate these hypothesized mechanisms more directly. We are also unable to distinguish the effects of different components of the FFCRA, which included a mix of financial incentives to states in addition to the continuous enrollment provision. This raises the possibility that future legislation using different financial incentives may lead to different outcomes.

Finally, we focused on insurance churn but did not examine any other consequences of the FFCRA, such as health care utilization or health outcomes. The broader range of implications of this national insurance stabilization event remain unknown. Going forward, we are particularly interested in using the FFCRA as a natural experiment to study the downstream effects of decreased insurance churn. We suspect that it will be most productive to focus on a subsample of individuals with certain conditions (such as cancer) where there are clinical reasons to suspect that insurance churn may be particularly consequential for patient health outcomes.

## Conclusions

In this study, the FFCRA was associated with a significantly decreased risk of losing health insurance, primarily by reducing Medicaid churn. The reduction in Medicaid churn was experienced across a broad range of demographic groups.

## Notes

Supplement 1.
eAppendix.
**eTable 1.** Summary Statistics Among Insured Individuals **eTable 2.** Summary Statistics Among Medicaid Beneficiaries **eTable 3.** Summary Statistics Among Individuals With Private, Employer-Provided Coverage **eTable 4.** Summary Statistics Among Individuals With Any Other Insurance Coverage **eTable 5.** Summary Statistics Among Uninsured Individuals **eTable 6.** Interrupted Time Series Estimates **eFigure 1.** Interrupted Time Series Estimates of the Share of Individuals Who Lost Any Coverage Within the Following 12 Months, by Age Group **eFigure 2.** Interrupted Time Series Estimates of the Share of Individuals Who Lost Any Coverage Within the Following 12 Months, by Race/Ethnicity **eFigure 3.** Interrupted Time Series Estimates of the Share of Individuals Who Lost Any Coverage Within the Following 12 Months, by Education Level **eFigure 4.** Interrupted Time Series Estimates of the Share of Individuals Who Lost Any Coverage Within the Following 12 Months, by Sex **eFigure 5.** Loss of Any Coverage, by Income (Percentage of Federal Poverty Level) **eFigure 6.** Loss of Any Coverage, by Employment Status **eFigure 7.** Loss of Any Coverage, by Health Status **eFigure 8.** Interrupted Time Series Estimates of the Share of Individuals Who Lost Coverage for 2 Consecutive Months Within the Following 12 Months **eFigure 9.** Interrupted Time Series Estimates of the Share of Individuals Who Lost Coverage Within the Following 12 Months, Excluding Partial Treatment Period **eFigure 10.** Difference-in-Difference Analysis: Medicaid vs Any Coverage **eTable 7.** Difference-in-Difference Regression Results for 12-Month Churn **eTable 8.** Interrupted Time Series Estimates for 6-Month Insurance Churn **eFigure 11.** Interrupted Time Series Estimates of the Share of Individuals Who Lost Coverage Within the Following 6 Months **eFigure 12.** Share Lost Coverage Within the Following 12 Months (Reg. Version, Unweighted) **eTable 9.** Interrupted Time Series (Unweighted) **eTable 10.** Difference-in-Difference Unweighted Regression Results (12 Month) **eFigure 13.** Unweighted Difference-in-Difference Analysis: Medicaid vs Private Coverage **eFigure 14.** Unweighted Difference-in-Difference Analysis: Medicaid vs Any Coverage **eFigure 15.** Share Lost Coverage Within the Following 12 Months (Reg. Version With Demographic Controls) **eFigure 16.** Difference-in-Difference Analysis With Demographic Controls: Medicaid vs Private Coverage **eFigure 17.** Difference-in-Difference Analysis With Demographic Controls: Medicaid vs Any Coverage **eTable 11.** Difference-in-Difference Regression Results With Demographic Controls (12 Month)

Supplement 2. Data Sharing Statement
