# Supplementary material for: Insurance Churn and the COVID-19 Pandemic
Source: JAMA Health Forum. 2025 Jun 27;6(6):e251467. doi: 10.1001/jamahealthforum.2025.1467 (PMC12205398; doi:10.1001/jamahealthforum.2025.1467)
Supplement: Supplement 1. — eAppendix. eTable 1. Summary Statistics Among Insured Individuals eTable 2. Summary Statistics Among Medicaid Beneficiaries eTable 3. Summary Statistics Among Individuals With Private, Employer-Provided Coverage eTable 4. Summary Statistics Among Individuals With Any Other Insurance Coverage eTable 5. Summary Statistics Among Uninsured Individuals eTable 6. Interrupted Time Series Estimates eFigure 1. Interrupted Time Series Estimates of the Share of Individuals Who Lost Any Coverage Within the Following 12 Months, by Age Group eFigure 2. Interrupted Time Series Estimates of the Share of Individuals Who Lost Any Coverage Within the Following 12 Months, by Race/Ethnicity eFigure 3. Interrupted Time Series Estimates of the Share of Individuals Who Lost Any Coverage Within the Following 12 Months, by Education Level eFigure 4. Interrupted Time Series Estimates of the Share of Individuals Who Lost Any Coverage Within the Following 12 Months, by Sex eFigure 5. Loss of Any Coverage, by Income (Percentage of Federal Poverty Level) eFigure 6. Loss of Any Coverage, by Employment Status eFigure 7. Loss of Any Coverage, by Health Status eFigure 8. Interrupted Time Series Estimates of the Share of Individuals Who Lost Coverage for 2 Consecutive Months Within the Following 12 Months eFigure 9. Interrupted Time Series Estimates of the Share of Individuals Who Lost Coverage Within the Following 12 Months, Excluding Partial Treatment Period eFigure 10. Difference-in-Difference Analysis: Medicaid vs Any Coverage eTable 7. Difference-in-Difference Regression Results for 12-Month Churn eTable 8. Interrupted Time Series Estimates for 6-Month Insurance Churn eFigure 11. Interrupted Time Series Estimates of the Share of Individuals Who Lost Coverage Within the Following 6 Months eFigure 12. Share Lost Coverage Within the Following 12 Months (Reg. Version, Unweighted) eTable 9. Interrupted Time Series (Unweighted) eTable 10. Difference-in-Difference Unweighted Regression Results (12 Month [file jamahealthforum-e251467-s001.pdf]

Supplemental Online Content

Shubeck SP, Crawford E, Notowidigdo MJ. Insurance churn and the COVID-19 pandemic. *JAMA Health Forum.* 2025;6(6):251467. doi:10.1001/jamahealthforum.2025.1467

eAppendix.

eTable 1. Summary Statistics Among Insured Individuals

eTable 2. Summary Statistics Among Medicaid Beneficiaries

eTable 3. Summary Statistics Among Individuals With Private, Employer-Provided Coverage

eTable 4. Summary Statistics Among Individuals With Any Other Insurance Coverage

eTable 5. Summary Statistics Among Uninsured Individuals

eTable 6. Interrupted Time Series Estimates

eFigure 1. Interrupted Time Series Estimates of the Share of Individuals Who Lost Any Coverage Within the Following 12 Months, by Age Group

eFigure 2. Interrupted Time Series Estimates of the Share of Individuals Who Lost Any Coverage Within the Following 12 Months, by Race/Ethnicity

eFigure 3. Interrupted Time Series Estimates of the Share of Individuals Who Lost Any Coverage Within the Following 12 Months, by Education Level

eFigure 4. Interrupted Time Series Estimates of the Share of Individuals Who Lost Any Coverage Within the Following 12 Months, by Sex

eFigure 5. Loss of Any Coverage, by Income (Percentage of Federal Poverty Level)

eFigure 6. Loss of Any Coverage, by Employment Status

eFigure 7. Loss of Any Coverage, by Health Status

eFigure 8. Interrupted Time Series Estimates of the Share of Individuals Who Lost Coverage for 2 Consecutive Months Within the Following 12 Months

eFigure 9. Interrupted Time Series Estimates of the Share of Individuals Who Lost Coverage Within the Following 12 Months, Excluding Partial Treatment Period

eFigure 10. Difference-in-Difference Analysis: Medicaid vs Any Coverage

eTable 7. Difference-in-Difference Regression Results for 12-Month Churn

eTable 8. Interrupted Time Series Estimates for 6-Month Insurance Churn

eFigure 11. Interrupted Time Series Estimates of the Share of Individuals Who Lost Coverage Within the Following 6 Months

eFigure 12. Share Lost Coverage Within the Following 12 Months (Reg. Version, Unweighted)

© 2025 Shubeck SP et al. *JAMA Health Forum*.

eTable 9. Interrupted Time Series (Unweighted)

eTable 10. Difference-in-Difference Unweighted Regression Results (12 Month)

eFigure 13. Unweighted Difference-in-Difference Analysis: Medicaid vs Private Coverage

eFigure 14. Unweighted Difference-in-Difference Analysis: Medicaid vs Any Coverage

eFigure 15. Share Lost Coverage Within the Following 12 Months (Reg. Version With Demographic Controls)

eFigure 16. Difference-in-Difference Analysis With Demographic Controls: Medicaid vs Private Coverage

eFigure 17. Difference-in-Difference Analysis With Demographic Controls: Medicaid vs Any Coverage

eTable 11. Difference-in-Difference Regression Results With Demographic Controls (12 Month)

This supplementary material has been provided by the authors to give readers additional information about their work.

© 2025 Shubeck SP et al. *JAMA Health Forum*.

eAppendix.

A MEPS Data Collection During the COVID-19 Pandemic

The MEPS data collection process was disrupted during the COVID-19 pandemic. The pandemic-related "shelter-in-place" policies meant that all in-person data collection was suspended in March 2020, which led to short-term changes in data collection and a move to telephone-based surveys during most of 2020. By Fall 2020, however, in-person interviews and door-to-door contacts were gradually resumed. Additionally, the interview rounds were modified, with additional interview rounds added to reduce non-response. The combination of adjustments led to maintaining sample sizes but a reduction in response rates during the early periods of the COVID-19 pandemic. As a result, we pool all of the data together in our main analysis and show sensitivity to excluding the estimated insurance churn rates during in the early months of the pandemic.

B Empirical Analysis

We use two research designs to identify and estimate the effects of the FFCRA: an interrupted time series model and a difference-in-difference model.

B.1 Interrupted Time Series Model

The interrupted time series model we estimate assumes that absent the FFCRA that insurance churn would have followed the same time trend as in the years prior to the FFCRA. That is, any structural break in the time trend after the FFCRA is implemented will be interpreted as being caused by the FFCRA.

We estimate the following linear spline regression:

$$(1) \quad y_{it} = \alpha_0 + \alpha_1 \cdot t + \beta_1 \cdot 1\{t \geq t_{FFCRA} - 12\} \cdot \left(t - (t_{FFCRA} - 12)\right) + \beta_2 \cdot 1\{t \geq t_{FFCRA}\} \cdot (t - t_{FFCRA}) + \varepsilon_{it}$$

The variable y_{it} in equation 1 is our measure of insurance churn which is whether an insured individual *i* in year-month *t* experiences any loss of insurance coverage within the next 12 months. The variable t_{FFCRA} indicates the year and month of the FFCRA, which is the beginning of the continuous coverage provisions. We define $t_{FFCRA} - 12$ to be the start of the "partially treated" time period, since after this time individuals' insurance churn will end up being at least partially affected by the FFCRA at some point over the next 12 months.

The main coefficients of interest are β_1 and β_2: β_1 represents the structural break at the beginning of the partial treatment time period, and β_2 represents the structural break at the start of the FFCRA relative to the trend at the beginning of the partial treatment period. To see how to translate the effects of the FFCRA into structural break coefficients, suppose that the FFCRA causes an immediate impact at time t_{FFCRA} and reduces the level of y_{it} by $-\delta$. Furthermore, assume that this effect gradually (and linearly) emerges during the partial treatment time period (i.e., between time $t_{FFCRA} - 12$ and time t_{FFCRA}). In this case, we would expect to estimate $\widehat{\beta_1} = -\frac{\delta}{12}$ (so that at $t = t_{FFCRA}$ the level of y_{it} has decreased by $-\delta$ relative to the pre-pandemic linear time trend, and $\widehat{\beta_2} = \frac{\delta}{12}$, so that y_{it} reverts back to the pre-FFCRA time trend after the one-time "level shift" caused by FFCRA.

B.2 Difference-in-Difference Model

We use a difference-in-difference model to identify the effects of the FFCRA on Medicaid churn by comparing the churn rates of Medicaid enrollees to the churn rates for the privately insured, before and after the FFCRA. We estimate an event study version of the difference-in-difference model, which is similar to a difference-in-difference model, but we specify a full set of event time dummies to capture flexible trends before and after the FFCRA. Specifically, we estimate the following event study regression model:

© 2025 Shubeck SP et al. *JAMA Health Forum*.

$$(2) \quad y_{it} = \gamma_0 + \gamma_1 \cdot 1\{\text{Medicaid}_i\} + \delta_t + \sum_{\tau \neq (t_{FFCRA}-12)-1} \lambda_t \cdot 1\{\text{Medicaid}_i\} \cdot 1\{t = \tau\} + \varepsilon_{it}$$

As in the interrupted time series analysis, the variable y_{it} in equation 2 is our primary measure of insurance churn. We control for time fixed effects (i.e., year-month indicators δ_t) as well as an indicator γ_1 for whether the individual begins on Medicaid (versus private insurance). The γ_t variables are the key coefficients of interest: these coefficients estimate the Medicaid churn rate relative to the churn rate for the privately insured for each year-month relative to the month before the beginning of the partial treatment period, $(t_{FFCRA} - 12)$.

To quantify the effects of the FFCRA on Medicaid enrollees relative the privately insured, we estimate the following difference-in-difference model:

$$\begin{aligned}(3) \quad y_{it} = {} & \gamma_0 + \gamma_1 \cdot 1\{\text{Medicaid}_i\} + \delta_t + \\ & \lambda_{\text{partial treatment period}} \cdot 1\{\text{Medicaid}_i\} \cdot 1\{t \geq t_{FFCRA} - 12\} \cdot 1\{t < t_{FFCRA}\} + \\ & \lambda_{FFCRA} \cdot 1\{\text{Medicaid}_i\} \cdot 1\{t \geq t_{FFCRA}\} + \varepsilon_{it}\end{aligned}$$

In the main text, we report the estimate of λ_{FFCRA} to quantify the average effect for the FFCRA.

© 2025 Shubeck SP et al. *JAMA Health Forum*.

eTable 1. Summary Statistics Among Insured Individuals

Characteristic	Total	Pre-FFCRA	Post-FFCRA
Total no[a]	77,557	66,354	11,203
Male[b]	48.80	48.67	49.32
Age Bin			
<18	29.61	29.77	28.95
18–44	38.99	38.71	40.12
45–64	31.41	31.52	30.93
Race/Ethnic Group[c]			
White, non-Hispanic	65.80	66.27	63.86
Black, non-Hispanic	14.18	14.06	14.67
Hispanic	20.02	19.67	21.48
Highest Level of Education[d]			
HS or Less	32.78	32.89	32.36
Some College or More	67.22	67.11	67.64

a. Sum of unique MEPSID indicators among individuals that had any type of insurance coverage in the beginning of their sample period. All statistics are calculated among individuals aged 2-64.
b. Age bins, sex, education levels, and insurance coverage are defined according to the response when first observed in the sample.
c. In cases of discrepancy, race/ethnic classification is defined as the modal response.
d. Statistics on educational attainment are calculated only among individuals aged 25 or older in the beginning of the survey.

© 2025 Shubeck SP et al. *JAMA Health Forum*.

eTable 2. Summary Statistics Among Medicaid Beneficiaries

Characteristic	Total	Pre-FFCRA	Post-FFCRA
Total no[a]	26,026	22,790	3,236
Male[b]	45.04	44.76	46.14
Age Bin			
<18	52.91	53.63	50.10
18–44	30.72	30.18	32.82
45–64	16.38	16.20	17.08
Race/Ethnic Group[c]			
White, non-Hispanic	43.28	43.06	44.12
Black, non-Hispanic	22.42	22.84	20.82
Hispanic	34.30	34.10	35.06
Highest Level of Education[d]			
HS or Less	61.61	61.74	61.15
Some College or More	38.39	38.26	38.85

a. Sum of unique MEPSID indicators among individuals enrolled in Medicaid in the beginning of their sample period. All statistics are calculated among individuals aged 2-64.

b. Age bins, sex, education levels, and insurance coverage are defined according to the response when first observed in the sample.

c. In cases of discrepancy, race/ethnic classification is defined as the modal response.

d. Statistics on educational attainment are calculated only among individuals aged 25 or older in the beginning of the survey.

© 2025 Shubeck SP et al. *JAMA Health Forum*.

eTable 3. Summary Statistics Among Individuals With Private, Employer-Provided Coverage

Characteristic	Total	Pre-FFCRA	Post-FFCRA
Total no[a]	45,435	38,461	6,974
Male[b]	50.06	49.95	50.54
Age Bin			
<18	23.63	23.70	23.37
18–44	42.21	41.94	43.35
45–64	34.16	34.36	33.28
Race/Ethnic Group[c]			
White, non-Hispanic	72.80	73.18	71.19
Black, non-Hispanic	11.54	11.36	12.31
Hispanic	15.66	15.46	16.50
Highest Level of Education[d]			
HS or Less	26.77	26.98	25.91
Some College or More	73.23	73.02	74.09

a. Sum of unique MEPSID indicators among individuals with private, employer-provided coverage in the beginning of their sample period. All statistics are calculated among individuals aged 2-64.
b. Age bins, sex, education levels, and insurance coverage are defined according to the response when first observed in the sample.
c. In cases of discrepancy, race/ethnic classification is defined as the modal response.
d. Statistics on educational attainment are calculated only among individuals aged 25 or older in the beginning of the survey.

© 2025 Shubeck SP et al. *JAMA Health Forum*.

eTable 4. Summary Statistics Among Individuals With Any Other Insurance Coverage

**Characteristic**	**Total**	**Pre-FFCRA**	**Post-FFCRA**
Total no[a]	9,875	8,283	1,592
Male[b]	48.32	48.15	48.96
Age Bin			
<18	15.88	15.81	16.13
18–44	34.96	34.46	36.88
45–64	49.16	49.73	46.99
Race/Ethnic Group[c]			
White, non-Hispanic	70.60	71.38	67.65
Black, non-Hispanic	14.22	14.14	14.51
Hispanic	15.18	14.48	17.84
Highest Level of Education[d]			
HS or Less	38.95	39.35	37.40
Some College or More	61.05	60.65	62.60

a. Sum of unique MEPSID indicators among individuals with any “other” coverage in the beginning of their sample period. “Other” coverage is defined as any health insurance coverage, excluding Medicaid and private, employer-provided coverage. All statistics are calculated among individuals aged 2-64.
b. Age bins, sex, education levels, and insurance coverage are defined according to the response when first observed in the sample.
c. In cases of discrepancy, race/ethnic classification is defined as the modal response.
d. Statistics on educational attainment are calculated only among individuals aged 25 or older in the beginning of the survey.

© 2025 Shubeck SP et al. *JAMA Health Forum*.

eTable 5. Summary Statistics Among Uninsured Individuals

Characteristic	Total	Pre-FFCRA	Post-FFCRA
Total no[a]	18,916	16,498	2,418
Male	53.90	54.00	53.50
Age Bin			
<18	15.22	15.48	14.15
18–44	59.23	59.82	56.82
45–64	25.55	24.69	29.03
Race/Ethnic Group			
White, non-Hispanic	48.98	48.99	48.98
Black, non-Hispanic	15.74	15.91	15.07
Hispanic	35.27	35.11	35.95
Highest Level of Education			
HS or Less	55.85	56.26	54.20
Some College or More	44.15	43.74	45.80

a. Sum of unique MEPSID indicators among that are uninsured in the beginning of their sample period. All statistics are calculated among individuals aged 2-64.
b. Age bins, sex, education levels, and insurance coverage are defined according to the response when first observed in the sample.
c. In cases of discrepancy, race/ethnic classification is defined as the modal response.
d. Statistics on educational attainment are calculated only among individuals aged 25 or older in the beginning of the survey.

© 2025 Shubeck SP et al. *JAMA Health Forum*.

eTable 6. Interrupted Time Series Estimates

Covariate[a]	Any Coverage	Medicaid	Private	Other
Pre FFCRA	-0.0000	0.0000	-0.0000	-0.0004***
	(0.0000)	(0.0001)	(0.0000)	(0.0001)
Partial Treatment	-0.0018***	-0.0056***	-0.0003	-0.0010***
	(0.0002)	(0.0003)	(0.0002)	(0.0004)
Post FFCRA	0.0018***	0.0064***	0.0001	0.0012***
	(0.0002)	(0.0004)	(0.0002)	(0.0004)
Constant	0.0794***	0.1268***	0.0569***	0.0799***
N	781,291,609	196,451,877	523,788,611	105,519,373

a. Reports the coefficient and standard errors from the Interrupted Time Series regression shown in Equation 1 for twelve-month churn. All regressions cluster standard errors at the year-month level and are weighted by inverse probability of selection into the sample, adjusted for non-response with post-stratification adjustments for age, race/ethnicity, and sex using the Census Bureau's population control totals.

© 2025 Shubeck SP et al. *JAMA Health Forum*.

D.2 Loss of Any Coverage, by Demographic

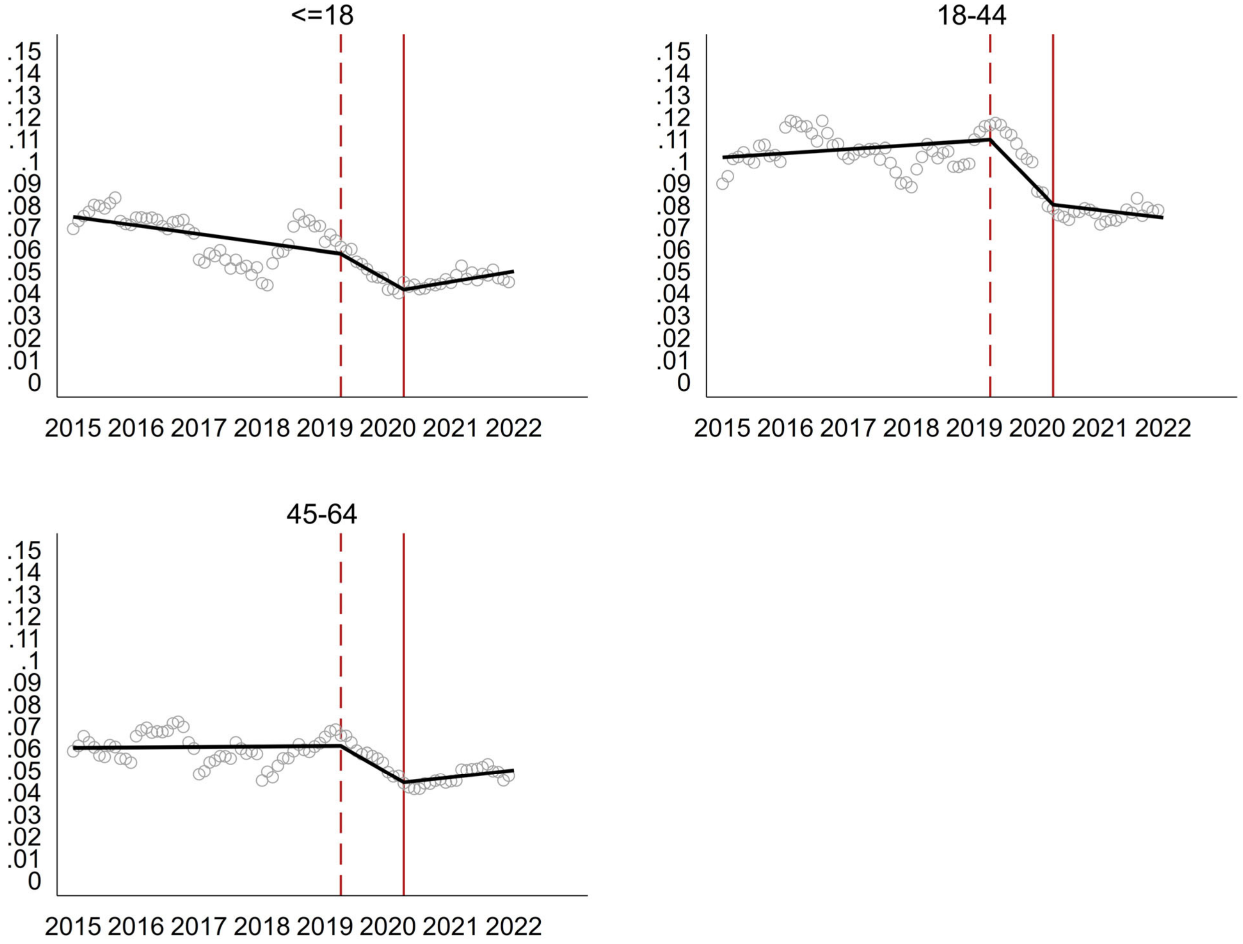

eFigure 1. Interrupted Time Series Estimates of the Share of Individuals Who Lost Any Coverage Within the Following 12 Months, by Age Group

The hollow dots display for each calendar month, among those with any insurance coverage, the share who ever experienced a period of being uninsured in the following twelve months. The solid lines are constructed by fitting a linear spline to the shares, allowing for separate slopes in the pre-treatment, partial treatment, and treatment periods. The panels show the rate of uninsurance among the following age groups: under 18, 18-44, and 45-64.
The red solid line indicates the date in which the Families First Coronavirus Response Act (FFCRA) was enacted: April 2020. The red dotted line shows the beginning of the “partially treated” sample: cohorts where future coverage overlaps with the passage of the FFCRA.

© 2025 Shubeck SP et al. *JAMA Health Forum*.

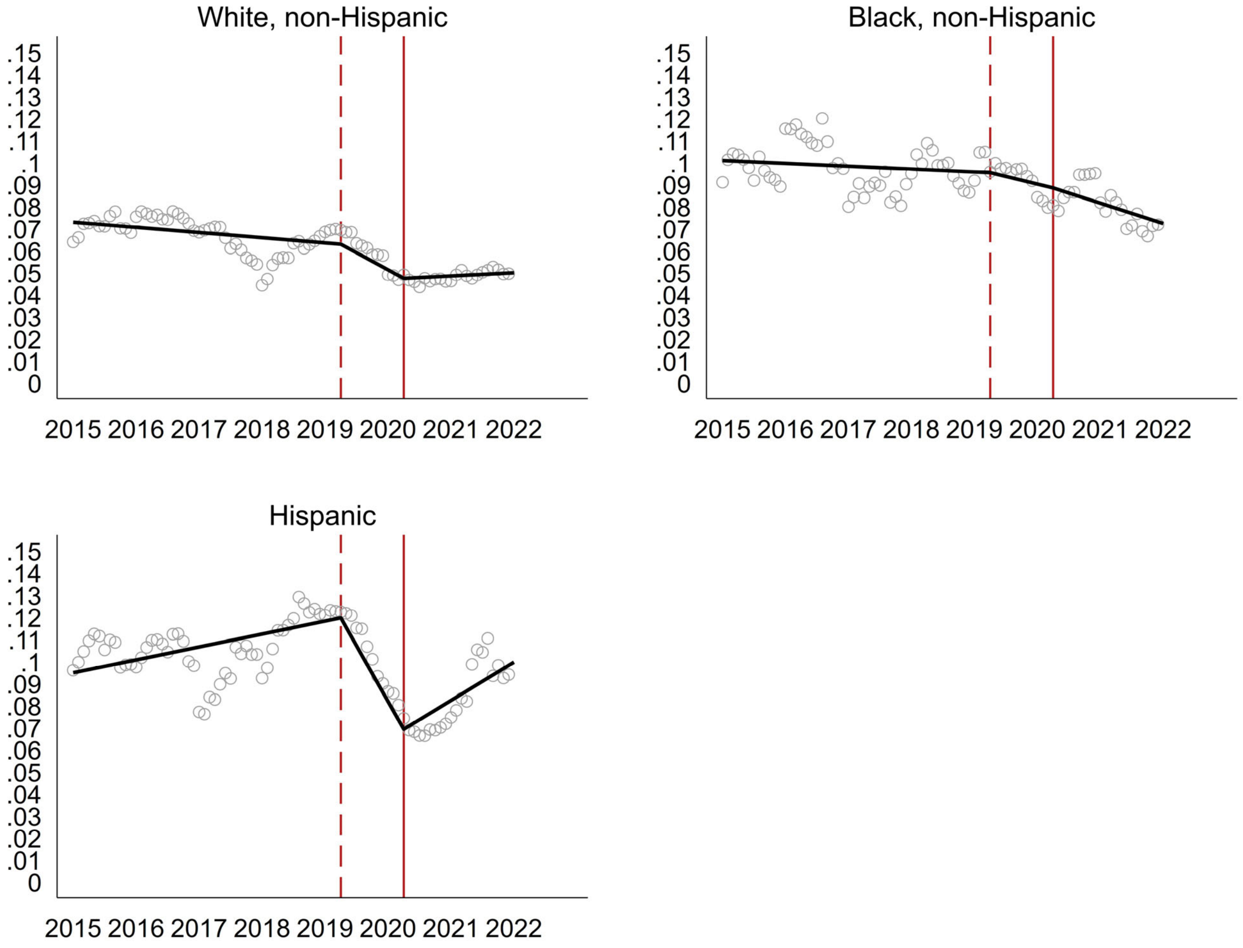

eFigure 2. Interrupted Time Series Estimates of the Share of Individuals Who Lost Any Coverage Within the Following 12 Months, by Race/Ethnicity

The hollow dots display for each calendar month, among those with any insurance coverage, the share who ever experienced a period of being uninsured in the following twelve months. The solid lines are constructed by fitting a linear spline to the shares, allowing for separate slopes in the pre-treatment, partial treatment, and treatment periods. The panels show the rate of uninsurance among the following race/ethnic groups: non-Hispanic white, non-Hispanic black, and Hispanic.
The red solid line indicates the date in which the Families First Coronavirus Response Act (FFCRA) was enacted: April 2020. The red dotted line shows the beginning of the "partially treated" sample: cohorts where future coverage overlaps with the passage of the FFCRA.

© 2025 Shubeck SP et al. *JAMA Health Forum*.

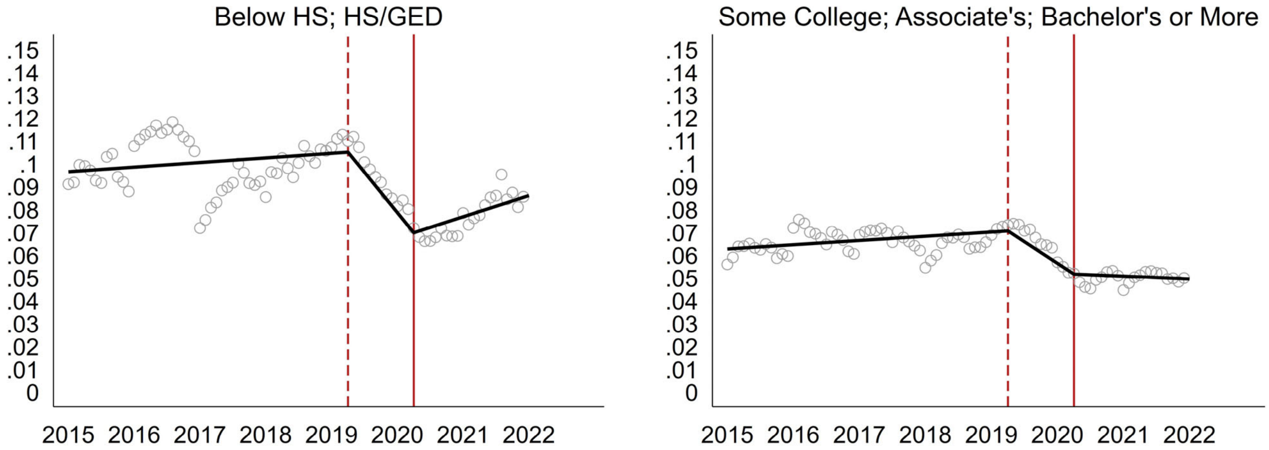

eFigure 3. Interrupted Time Series Estimates of the Share of Individuals Who Lost Any Coverage Within the Following 12 Months, by Education Level

The hollow dots display for each calendar month, among those with any insurance coverage, the share who ever experienced a period of being uninsured in the following twelve months. The solid lines are constructed by fitting a linear spline to the shares, allowing for separate slopes in the pre-treatment, partial treatment, and treatment periods. The graphs show the rate of uninsurance among the following education groups: below HS, HS/GED; Some College, Associate's, Bachelor's degree or higher.

The red solid line indicates the date in which the Families First Coronavirus Response Act (FFCRA) was enacted: April 2020. The red dotted line shows the beginning of the "partially treated" sample: cohorts where future coverage overlaps with the passage of the FFCRA.

© 2025 Shubeck SP et al. *JAMA Health Forum*.

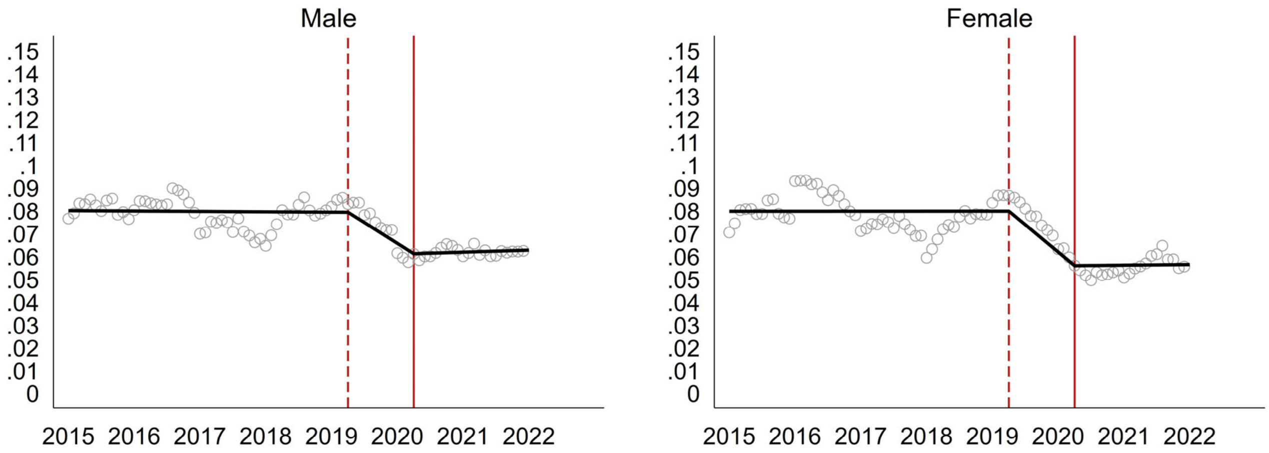

eFigure 4. Interrupted Time Series Estimates of the Share of Individuals Who Lost Any Coverage Within the Following 12 Months, by Sex

The hollow dots display for each calendar month, among those with any insurance coverage, the share who ever experienced a period of being uninsured in the following twelve months. The solid lines are constructed by fitting a linear spline to the shares, allowing for separate slopes in the pre-treatment, partial treatment, and treatment periods. The graphs show the rate of uninsurance separately among men and women.

The red solid line indicates the date in which the Families First Coronavirus Response Act (FFCRA) was enacted: April 2020. The red dotted line shows the beginning of the "partially treated" sample: cohorts where future coverage overlaps with the passage of the FFCRA.

© 2025 Shubeck SP et al. *JAMA Health Forum*.

D.3 Robustness Check: Loss of any Coverage, by Socioeconomic Status.

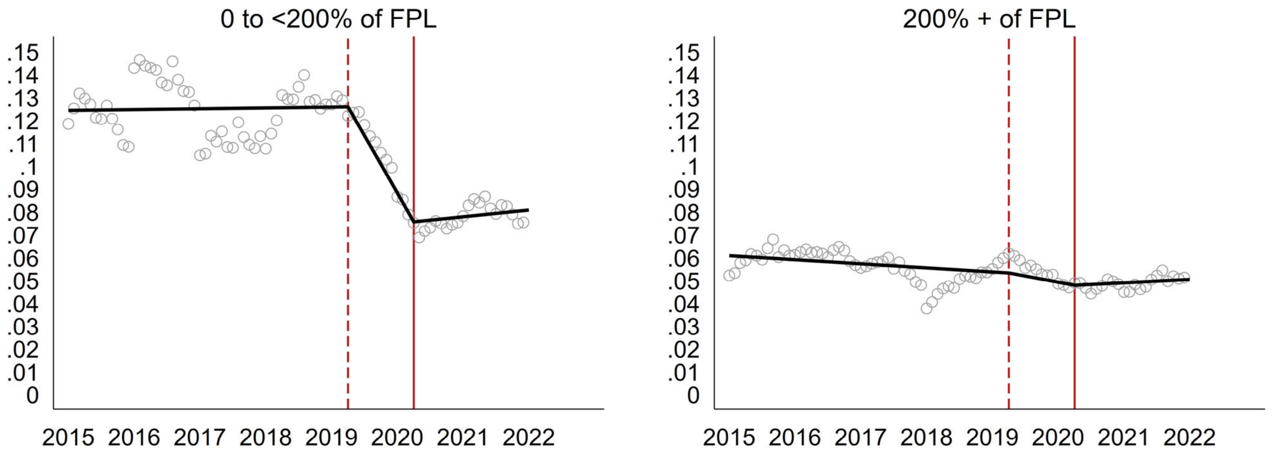

eFigure 5. Loss of Any Coverage, by Income (Percentage of Federal Poverty Level)

Displays for each calendar month, among those with any insurance coverage, the share who ever experienced a period of being uninsured in the following twelve months. The red solid line indicates the date in which the Families First Coronavirus Response Act (FFCRA) was enacted: April 2020. The red dotted line shows the "partially treated" sample: cohorts where future coverage overlaps with the passage of the FFCRA.

The graphs show the rate of churn by total family personal income as a percentage of the federal poverty line.

© 2025 Shubeck SP et al. *JAMA Health Forum*.

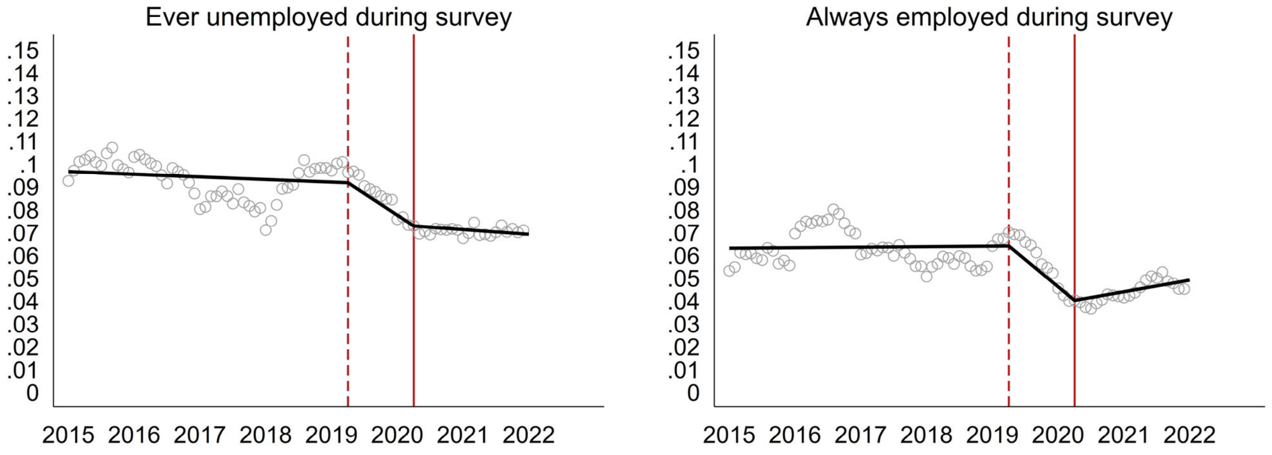

eFigure 6. Loss of Any Coverage, by Employment Status

Displays for each calendar month, among those with any insurance coverage, the share who ever experienced a period of being uninsured in the following twelve months. The red solid line indicates the date in which the Families First Coronavirus Response Act (FFCRA) was enacted: April 2020. The red dotted line shows the “partially treated” sample: cohorts where future coverage overlaps with the passage of the FFCRA.

The graphs show the rate of churn separately by annual employment status. We define an individual-year as ‘employed’, if, during any of the interviews (rounds) occurring in that year, they were concurrently employed. We define an individual as ‘unemployed’, if, during all of the interviews occurring in that year, they were never concurrently employed.

© 2025 Shubeck SP et al. *JAMA Health Forum*.

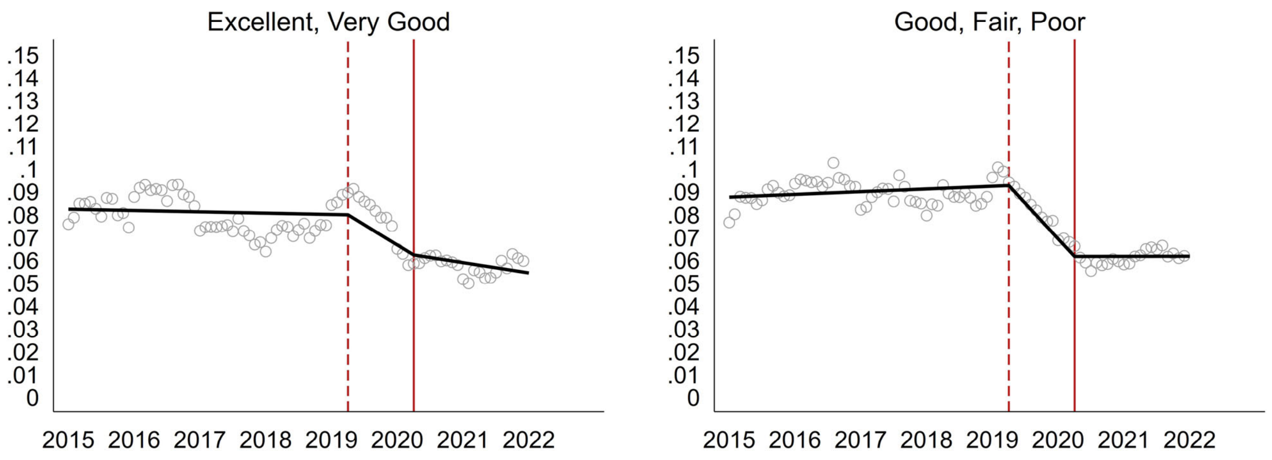

eFigure 7. Loss of Any Coverage, by Health Status

Displays for each calendar month, among those with any insurance coverage, the share who ever experienced a period of being uninsured in the following twelve months. The red solid line indicates the date in which the Families First Coronavirus Response Act (FFCRA) was enacted: April 2020. The red dotted line shows the “partially treated” sample: cohorts where future coverage overlaps with the passage of the FFCRA.

The graphs show the rate of churn by self-reported health status.

All means and regression results are weighted by the Self-Administered Questionnaire weight. For each year, the sum of these weights is equal to that year’s civilian, non-institutionalized U.S. population aged 18 and older, adjusted for non-response.

© 2025 Shubeck SP et al. *JAMA Health Forum*.

D.4 Robustness Check: Measurement Error

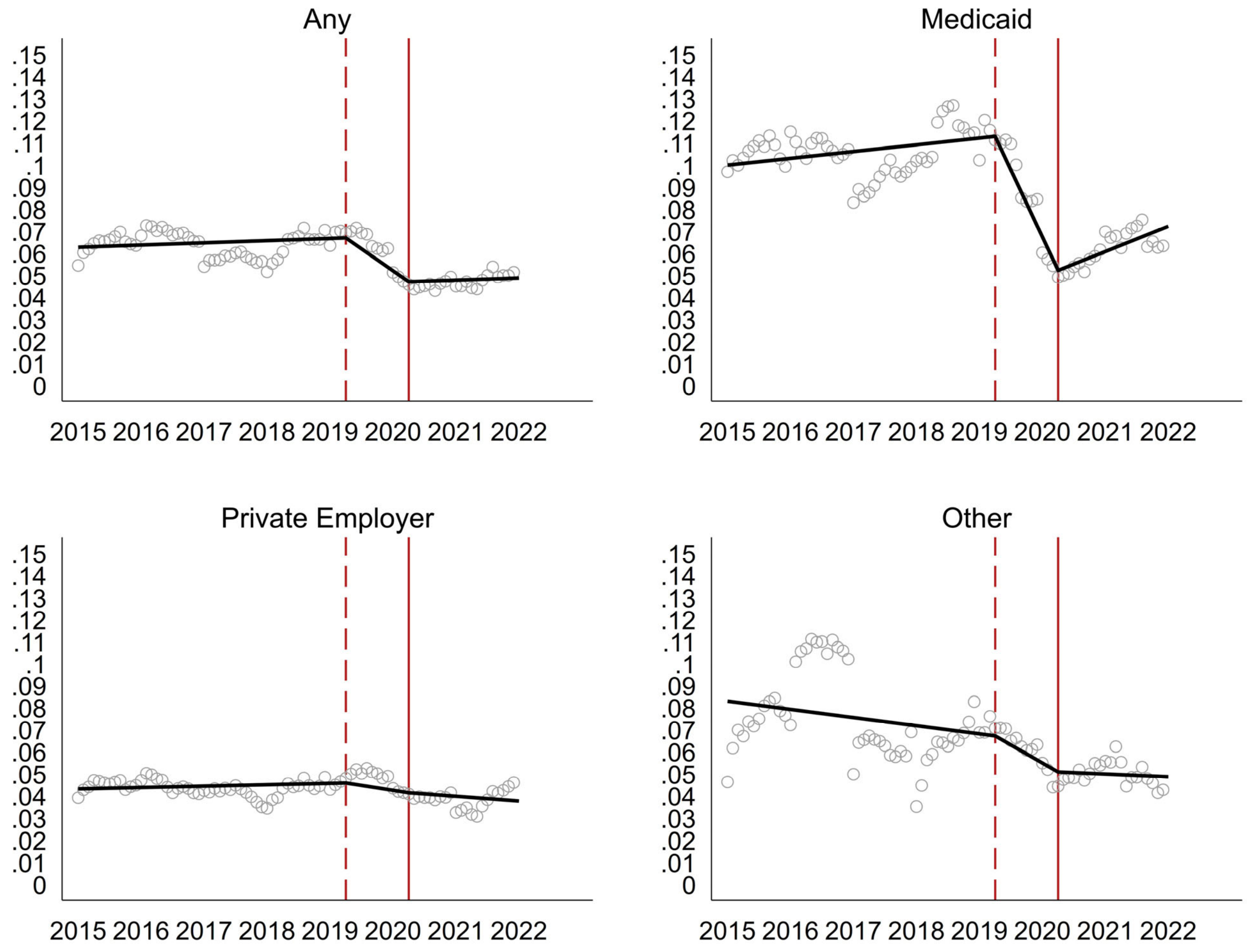

eFigure 8. Interrupted Time Series Estimates of the Share of Individuals Who Lost Coverage for 2 Consecutive Months Within the Following 12 Months

The hollow dots display for each calendar month, among those with any coverage, Medicaid, private employer-provided coverage, or other coverage, the share who ever experienced a period of two consecutive months of uninsurance in the following twelve months.
The solid lines are constructed by fitting a linear spline to the shares, allowing for separate slopes in the pre-treatment, partial treatment, and treatment periods.
The red solid line indicates the date in which the Families First Coronavirus Response Act (FFCRA) was enacted: April 2020. The red dotted line shows the beginning of the “partially treated” sample: cohorts where future coverage overlaps with the passage of the FFCRA.

© 2025 Shubeck SP et al. *JAMA Health Forum*.

D.5 **Robustness Check: Exclude Partial Treatment Period**

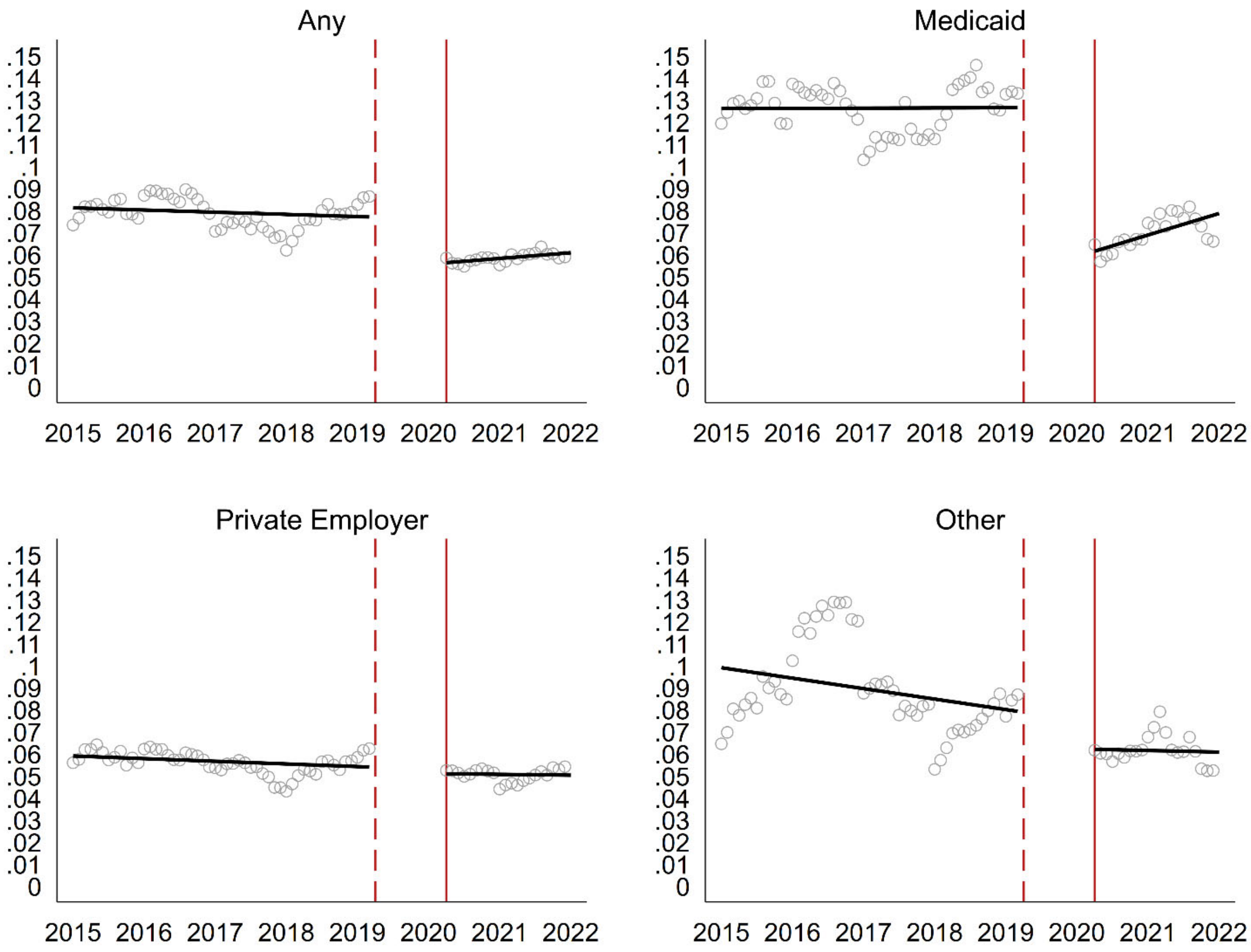

eFigure 9. Interrupted Time Series Estimates of the Share of Individuals Who Lost Coverage Within the Following 12 Months, Excluding Partial Treatment Period

The hollow dots display for each calendar month, among those with any coverage, Medicaid, private employer-provided coverage, or other coverage, the share who ever experienced a period of uninsurance in the following twelve months. The solid lines are constructed by separately fitting linear splines to the shares in the pre-treatment and full treatment periods, excluding 12 months of data from April 2019 up to April 2020.

The red solid line indicates the date in which the Families First Coronavirus Response Act (FFCRA) was enacted: April 2020. The red dotted line shows the beginning of the “partially treated” sample: cohorts where future coverage overlaps with the passage of the FFCRA.

E Difference-in-Difference Analysis

E.1 Overall

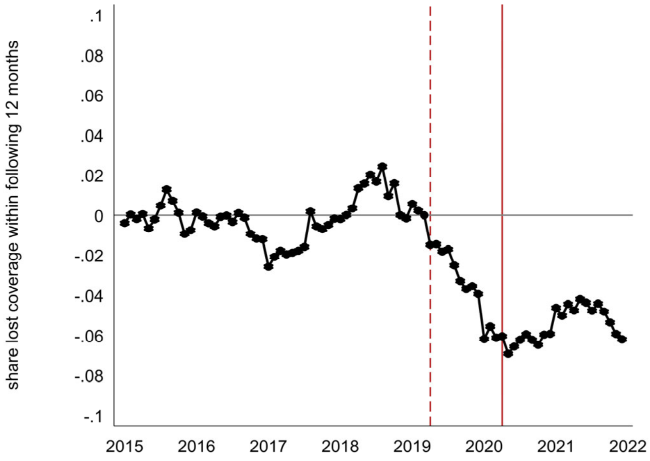

eFigure 10. Difference-in-Difference Analysis: Medicaid vs Any Coverage

The dots display the normalized difference-in-difference coefficient, where the outcome variable is twelve-month churn. The treatment group is comprised of all those with Medicaid coverage, and the comparison group is all those with any type of health insurance coverage other than Medicaid, including private coverage (from employer or union group, from other group or non-group insurance, from a federal or state exchange, or from an unknown source) and public coverage (Medicare or Tricare).

The red solid line indicates the date in which the Families First Coronavirus Response Act (FFCRA) was enacted: April 2020. The red dotted line shows the beginning of the "partially treated" sample: cohorts where future coverage overlaps with the passage of the FFCRA

© 2025 Shubeck SP et al. *JAMA Health Forum*.

eTable 7. Difference-in-Difference Regression Results for 12-Month Churn

**Covariate**[a]	**Private Coverage**[b]	**Any Coverage**[c]
Medicaid × Partial Treatment	-0.0320***	-0.0302***
	(0.0052)	(0.0054)
Medicaid × Treatment	-0.0551***	-0.0549***
	(0.0024)	(0.0023)
N	755,516	830,969

a. Reports the difference-in-difference coefficients $\lambda_{partial}$ and λ_{FFCRA}, respectively, as shown in Equation 3, for the outcome of the twelve-month churn rate. All regressions cluster standard errors at the year-month level and are weighted by inverse probability of selection into the sample, adjusted for non-response with post-stratification adjustments for age, race/ethnicity, and sex using the Census Bureau's population control totals.

b. Defines the Medicaid comparison group as those with private coverage provided by from an employer or union group.

c. Defines the Medicaid comparison group as those with any health insurance coverage other than Medicaid, including private coverage (from employer or union group, from other group or non-group insurance, from a federal or state exchange, or from an unknown source) and public coverage (Medicare or Tricare).

© 2025 Shubeck SP et al. *JAMA Health Forum*.

F Robustness: Six Month Churn

eTable 8. Interrupted Time Series Estimates for 6-Month Insurance Churn

**Covariate[a]**	**Any Coverage**	**Medicaid**	**Private**	**Other**
Pre FFCRA	-0.0000	0.0000	-0.0000	-0.0000
	(0.0000)	(0.0000)	(0.0000)	(0.0001)
Partial Treatment	-0.0017***	-0.0057***	-0.0001	-0.0020***
	(0.0002)	(0.0004)	(0.0002)	(0.0005)
Post FFCRA	0.0018***	0.0060***	0.0001	0.0019***
	(0.0002)	(0.0004)	(0.0002)	(0.0005)
Constant	0.0414***	0.0689***	0.0282***	0.0438***
N	1,266,417,343	313,311,584	853,178,514	171,829,495

a. Reports the coefficient and standard errors from the Interrupted Time Series regression shown in Equation 1 for six-month churn. All regressions cluster standard errors at the year-month level and are weighted by inverse probability of selection into the sample, adjusted for non-response with post-stratification adjustments for age, race/ethnicity, and sex using the Census Bureau's population control totals.

© 2025 Shubeck SP et al. *JAMA Health Forum*.

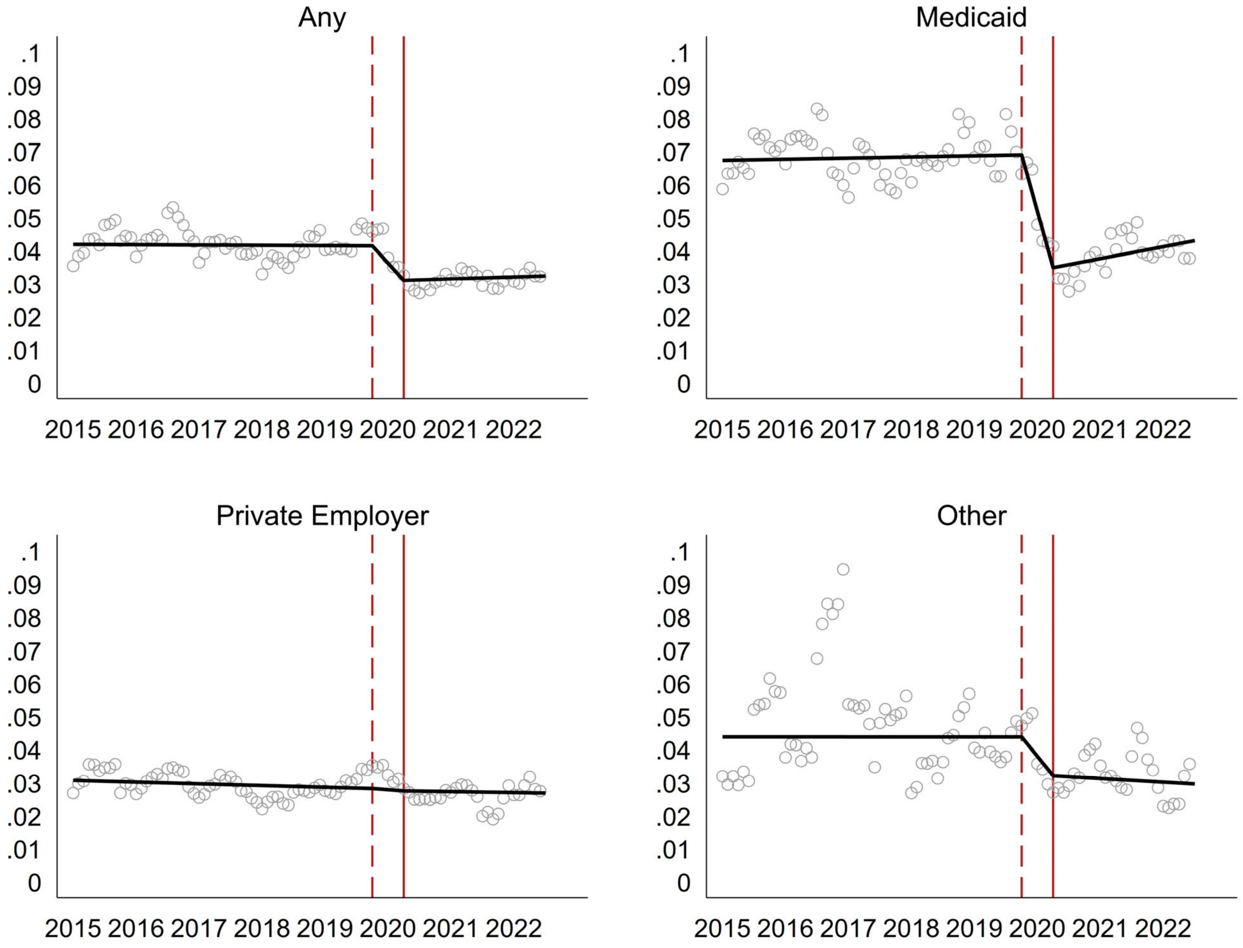

eFigure 11. Interrupted Time Series Estimates of the Share of Individuals Who Lost Coverage Within the Following 6 Months

The hollow dots display for each calendar month, among those with any coverage, Medicaid, private employer-provided coverage, or other coverage, the share who ever experienced a period of being uninsured in the following six months. The solid lines are constructed by fitting a linear spline to the shares, allowing for separate slopes in the pre-treatment, partial treatment, and treatment periods.
The red solid line indicates the date in which the Families First Coronavirus Response Act (FFCRA) was enacted: April 2020. The red dotted line shows the beginning of the "partially treated" sample: cohorts where future coverage overlaps with the passage of the FFCRA.

© 2025 Shubeck SP et al. *JAMA Health Forum*.

G Robustness: Unweighted

G.1 ITS

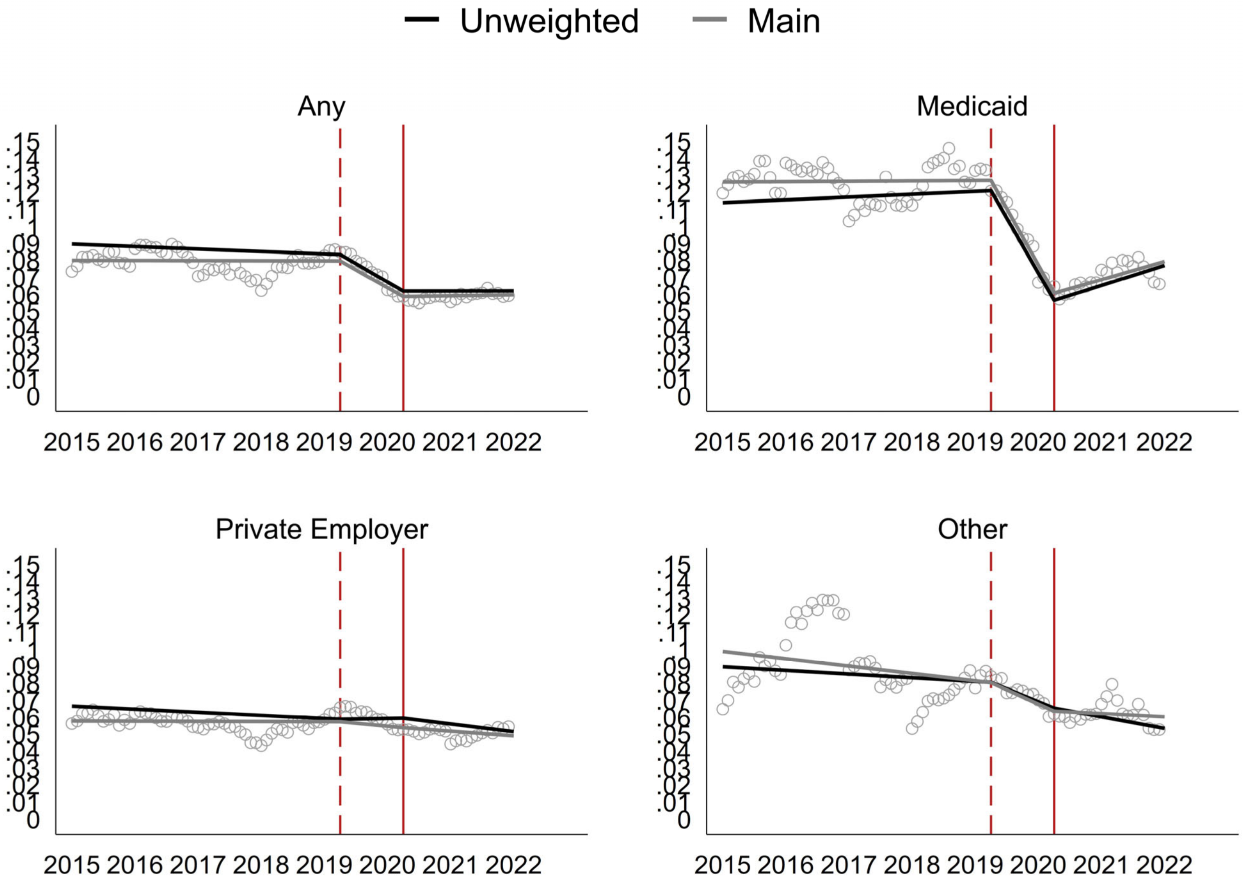

eFigure 12. Share Lost Coverage Within the Following 12 Months (Reg. Version, Unweighted)

The hollow dots display for each calendar month, among those with any coverage, Medicaid, private employer-provided coverage, or other coverage, the share who ever experienced a period of being uninsured in the following twelve months. The black lines are constructed by fitting an unweighted linear spline to the shares, allowing for separate slopes in the pre-treatment, partial treatment, and treatment periods. The gray lines show the fitted values for the main (i.e. weighted) regression, also shown in Figure 2.

The red solid line indicates the date in which the Families First Coronavirus Response Act (FFCRA) was enacted: April 2020. The red dotted line shows the beginning of the “partially treated” sample: cohorts where future coverage overlaps with the passage of the FFCRA.

© 2025 Shubeck SP et al. *JAMA Health Forum*.

eTable 9. Interrupted Time Series (Unweighted)

**Covariate**[a]	**Any Coverage**	**Medicaid**	**Private**	**Other**
Pre FFCRA	-0.0001***	0.0001***	-0.0002***	-0.0002**
	(0.0000)	(0.0000)	(0.0000)	(0.0001)
Partial Treatment	-0.0017***	-0.0055***	0.0002	-0.0011***
	(0.0002)	(0.0002)	(0.0002)	(0.0003)
Post FFCRA	0.0018***	0.0064***	-0.0004**	0.0007*
	(0.0002)	(0.0003)	(0.0002)	(0.0004)
Constant	0.0832***	0.1207***	0.0584***	0.0801***
N	839,447	290,143	485,569	117,020

a. Reports the coefficient and standard errors from the Interrupted Time Series regression shown in Equation 1 for twelve-month churn. All regressions cluster standard errors at the year-month level and are weighted by inverse probability of selection into the sample, adjusted for non-response with post-stratification adjustments for age, race/ethnicity, and sex using the Census Bureau’s population control totals. Regressions are unweighted. The coefficients for the main (i.e. weighted) regression are shown in eTable 6.

© 2025 Shubeck SP et al. *JAMA Health Forum*.

G.2 DiD

eTable 10. Difference-in-Difference Unweighted Regression Results (12 Month)

Covariate[a]	Private Coverage[b]	Any Coverage[c]
Medicaid × Partial Treatment	-0.0242***	-0.0211***
	(0.0056)	(0.0057)
Medicaid × Treatment	-0.0461***	-0.0432***
	(0.0023)	(0.0024)
N	762,769	839,442

a. Reports the difference-in-difference coefficients $\lambda_{partial}$ and λ_{FFCRA}, respectively, as shown in Equation 3, for the outcome of the twelve-month churn rate. All regressions cluster standard errors at the year-month level and are unweighted. The regression coefficients for the main (i.e weighted) specification are shown in eTable 7.

b. Defines the Medicaid comparison group as those with private coverage provided by from an employer or union group.

c. Defines the Medicaid comparison group as those with any type any health insurance coverage other than Medicaid, including private coverage(from employer or union group, from other group or non-group insurance, from a federal or state exchange, or from an unknown source) and public coverage (Medicare or Tricare).

© 2025 Shubeck SP et al. *JAMA Health Forum*.

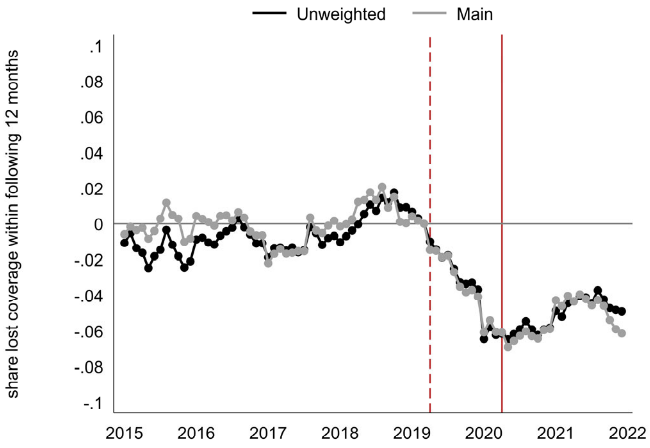

eFigure 13. Unweighted Difference-in-Difference Analysis: Medicaid vs Private Coverage

Displays the normalized difference-in-difference coefficients, where the outcome variable is twelve-month churn. The black dots displays the coefficients from the unweighted regression and the gray dots display the coefficients from the main (i.e. weighted) regression. The coefficients from the main regression are also shown in Figure 3.

The treatment group is comprised of all those with Medicaid coverage, and the comparison group is all those private coverage from an employer or union group.

The red solid line indicates the date in which the Families First Coronavirus Response Act (FFCRA) was enacted: April 2020. The red dotted line shows the beginning of the “partially treated” sample: cohorts where future coverage overlaps with the passage of the FFCRA.

© 2025 Shubeck SP et al. *JAMA Health Forum*.

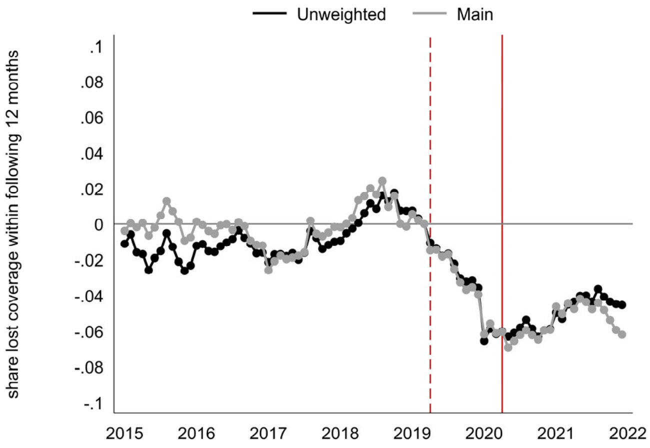

eFigure 14. Unweighted Difference-in-Difference Analysis: Medicaid vs Any Coverage

The dots display the normalized difference-in-difference coefficients, where the outcome variable is twelve-month churn. The black dots displays the coefficients from the unweighted regression, and the gray dots display the coefficients from the main (i.e. weighted) regression. The coefficients from the main regression are also shown in eFigure 10.

The treatment group is comprised of all those with Medicaid coverage, and the comparison group is all those with any type any health insurance coverage other than Medicaid, including private coverage (from employer or union group, from other group or non-group insurance, from a federal or state exchange, or from an unknown source) and public coverage (Medicare or Tricare).

The red solid line indicates the date in which the Families First Coronavirus Response Act (FFCRA) was enacted: April 2020. The red dotted line shows the beginning of the “partially treated” sample: cohorts where future coverage overlaps with the passage of the FFCRA.

© 2025 Shubeck SP et al. *JAMA Health Forum*.

H Robustness: Demographic Controls

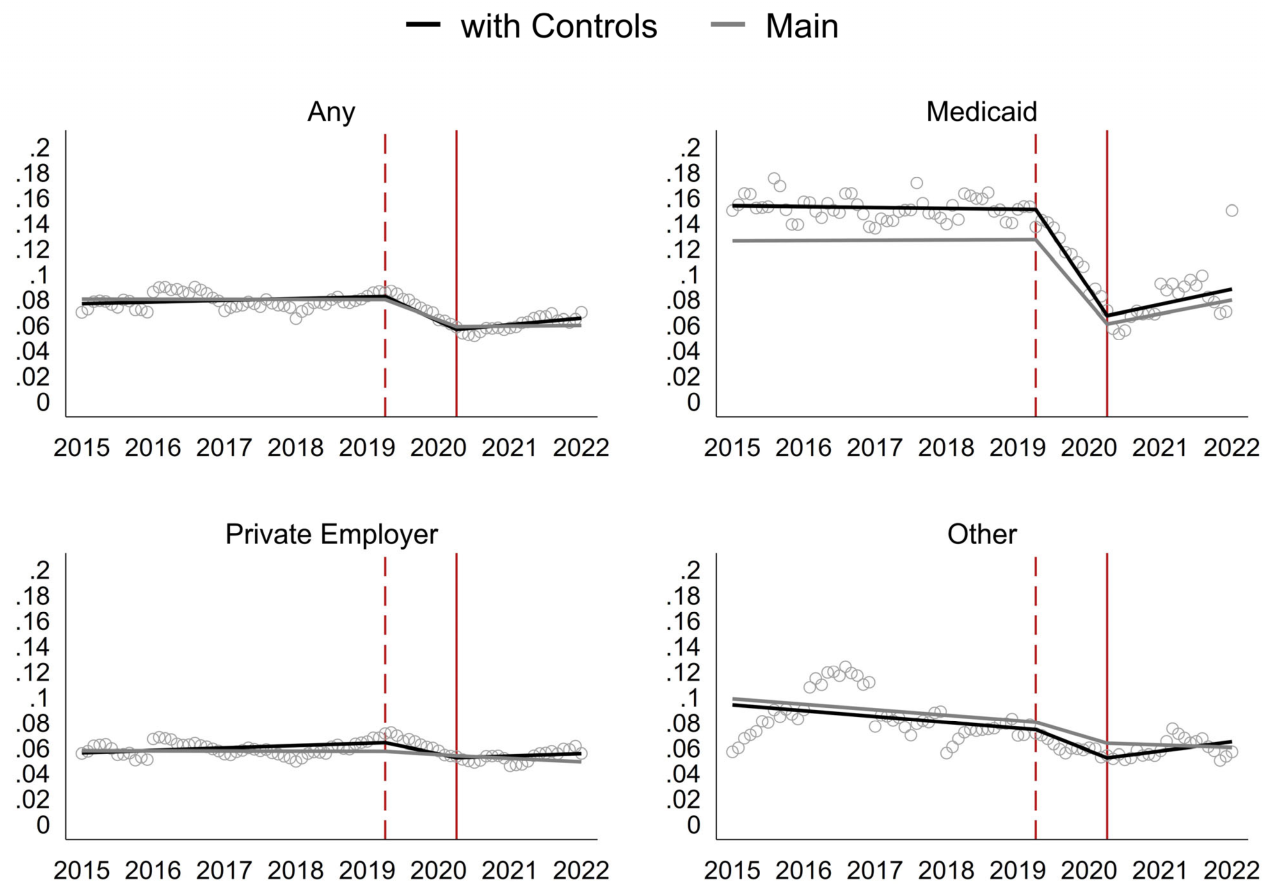

eFigure 15. Share Lost Coverage Within the Following 12 Months (Reg. Version With Demographic Controls)

The hollow dots display for each calendar month, among those with any coverage, Medicaid, private employer-provided coverage, or other coverage, the share who ever experienced a period of being uninsured in the following twelve months. The black lines are constructed by fitting an linear spline to the shares, controlling for age groups, race/ethnicity, sex, and education level, and allowing for separate slopes in the pre-treatment, partial treatment, and treatment periods. The gray lines show the fitted values for the main regression (i.e. without demographic controls), also shown in Figure 2.

The red solid line indicates the date in which the Families First Coronavirus Response Act (FFCRA) was enacted: April 2020. The red dotted line shows the beginning of the "partially treated" sample: cohorts where future coverage overlaps with the passage of the FFCRA.

© 2025 Shubeck SP et al. *JAMA Health Forum*.

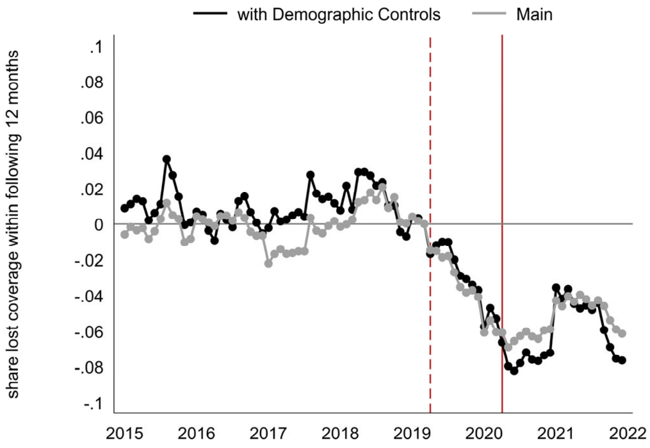

eFigure 16. Difference-in-Difference Analysis With Demographic Controls: Medicaid vs Private Coverage

Displays the normalized difference-in-difference coefficients, where the outcome variable is twelve-month churn. The black dots displays the coefficients from a regression with controls for age groups, race/ethnicity, sex, and education level. The gray dots display the coefficients from the main regression (i.e. without demographic controls). The coefficients from the main regression are also shown in Figure 3.

The treatment group is comprised of all those with Medicaid coverage, and the comparison group is all those private coverage from an employer or union group.

The red solid line indicates the date in which the Families First Coronavirus Response Act (FFCRA) was enacted: April 2020. The red dotted line shows the beginning of the "partially treated" sample: cohorts where future coverage overlaps with the passage of the FFCRA.

© 2025 Shubeck SP et al. *JAMA Health Forum*.

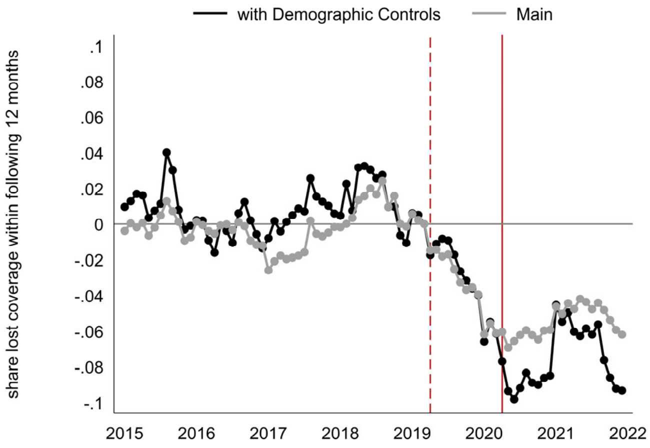

eFigure 17. Difference-in-Difference Analysis With Demographic Controls: Medicaid vs Any Coverage

Displays the normalized difference-in-difference coefficients, where the outcome variable is twelve-month churn. The black dots displays the coefficients from a regression with controls for age groups, race/ethnicity, sex, and education level. The gray dots display the coefficients from the main regression (i.e. without demographic controls). The coefficients from the main regression are also shown in eFigure 10.

The treatment group is comprised of all those with Medicaid coverage, and the comparison group is all those with any type any health insurance coverage other than Medicaid, including private coverage (from employer or union group, from other group or non-group insurance, from a federal or state exchange, or from an unknown source) and public coverage (Medicare or Tricare).

The red solid line indicates the date in which the Families First Coronavirus Response Act (FFCRA) was enacted: April 2020. The red dotted line shows the beginning of the “partially treated” sample: cohorts where future coverage overlaps with the passage of the FFCRA.

© 2025 Shubeck SP et al. *JAMA Health Forum*.

eTable 11. Difference-in-Difference Regression Results With Demographic Controls (12 Month)

Month)

**Covariate**[a]	**Private Coverage**[b]	**Any Coverage**[c]
Medicaid × Partial Treatment	-0.0165***	-0.0374***
	(0.0043)	(0.0061)
Medicaid × Treatment	-0.0383***	-0.0862***
	(0.0019)	(0.0038)
N	619,184	449,272

a. Reports the difference-in-difference coefficients $\lambda_{partial}$ and λ_{FFCRA}, respectively, as shown in Equation 3, for the outcome of the twelve-month churn rate. All regressions cluster standard errors at the the year-month level and control for age groups, race/ethnicity, sex, and education level. The regression coefficients for the main specification (i.e without demographic controls) are shown in eTable 7.

b. Defines the Medicaid comparison group as those with private coverage provided by from an employer or union group.

c. Defines the Medicaid comparison group as those with any type any health insurance coverage other than Medicaid, including private coverage(from employer or union group, from other group or non-group insurance, from a federal or state exchange, or from an unknown source) and public coverage (Medicare or Tricare).

© 2025 Shubeck SP et al. *JAMA Health Forum*.
