# Supplementary material for: Insurance Churn and the COVID-19 Pandemic
Source: JAMA Health Forum. 2025 Jun 27;6(6):e251467. doi: 10.1001/jamahealthforum.2025.1467 (PMC12205398; doi:10.1001/jamahealthforum.2025.1467)
Supplement: Supplement 2. — Data Sharing Statement [file jamahealthforum-e251467-s002.pdf]

Data Sharing Statement

Shubeck. Insurance Churn and the COVID-19 Pandemic. *JAMA Health Forum*. Published June 27, 2025. doi:10.1001/jamahealthforum.2025.1467

Data
Data available: Yes
Data types: Deidentified participant data
How to access data: Data publicly available - https://meps.ahrq.gov/mepsweb/
When available: With publication

Supporting Documents
Document types: Statistical/analytic code
How to access documents: shubeck@bsd.uchicago.edu
When available: With publication

Additional Information
Who can access the data: Anyone requesting the data
Types of analyses: Any purpose
Mechanisms of data availability: Data is publicly available - https://meps.ahrq.gov/mepsweb/
